# Highly Porous Amorphous Calcium Phosphate for Drug Delivery and Bio-Medical Applications

**DOI:** 10.3390/nano10010020

**Published:** 2019-12-19

**Authors:** Rui Sun, Michelle Åhlén, Cheuk-Wai Tai, Éva G. Bajnóczi, Fenne de Kleijne, Natalia Ferraz, Ingmar Persson, Maria Strømme, Ocean Cheung

**Affiliations:** 1 Division of Nanotechnology and Functional Materials, Department of Engineering Sciences, Uppsala University, SE-751 21 Uppsala, Sweden; rui.sun@angstrom.uu.se (R.S.); michelle.ahlen@angstrom.uu.se (M.Å.); fenne.dekleijne@student.fontys.nl (F.d.K.); natalia.ferraz@angstrom.uu.se (N.F.); 2 Department of Materials and Environmental Chemistry, Stockholm University, SE-106 91 Stockholm, Sweden; cheuk-wai.tai@mmk.su.se; 3 Department of Molecular Sciences, Swedish University of Agricultural Sciences, SE-750 07 Uppsala, Sweden; eva.bajnoczi@slu.se (É.G.B.); ingmar.persson@slu.se (I.P.)

**Keywords:** amorphous calcium phosphate, porous materials, cytocompatibility, drug carrier, bisphosphonate

## Abstract

Amorphous calcium phosphate (ACP) has shown significant effects on the biomineralization and promising applications in bio-medicine. However, the limited stability and porosity of ACP material restrict its practical applications. A storage stable highly porous ACP with Brunauer–Emmett–Teller surface area of over 400 m^2^/g was synthesized by introducing phosphoric acid to a methanol suspension containing amorphous calcium carbonate nanoparticles. Electron microscopy revealed that the porous ACP was constructed with aggregated ACP nanoparticles with dimensions of several nanometers. Large angle X-ray scattering revealed a short-range atomic order of <20 Å in the ACP nanoparticles. The synthesized ACP demonstrated long-term stability and did not crystallize even after storage for over 14 months in air. The stability of the ACP in water and an α-MEM cell culture medium were also examined. The stability of ACP could be tuned by adjusting its chemical composition. The ACP synthesized in this work was cytocompatible and acted as drug carriers for the bisphosphonate drug alendronate (AL) in vitro. AL-loaded ACP released ~25% of the loaded AL in the first 22 days. These properties make ACP a promising candidate material for potential application in biomedical fields such as drug delivery and bone healing.

## 1. Introduction

Calcium phosphate (CaP) is a group of materials and minerals containing calcium and phosphate ions often with one or two additional inorganic ions such as hydroxide, fluoride, and carbonate. CaP is the main mineral component of bones and teeth in vertebrates and an important biomaterial that has been widely used in drug/protein delivery and bone regeneration due to its functionality and biocompatibility [1,2,3]. Amorphous calcium phosphate (ACP) is one of the polymorphs of CaP. ACP typically exists as an intermediate phase that forms during the precipitation of CaP and is known to be an essential precursor in the formation of bones in vertebrates [4,5,6]. ACP in its natural solid form typically comprises an assembly of ACP nanoparticles. ACP with specific surface area of up to around 300 m^2^/g has been reported previously [7]. ACP has been considered for drug delivery due to its high capacity for drug loading and controlled release [8,9]. In addition, ACP is bioactive, with better biodegradability than crystalline CaP and with the ability to promote osteoblast adhesion [10] and osteconductivity [11]. These properties make ACP a promising candidate material for bone regeneration [9,12,13,14], in applications such as bone cements [15] and bio-ceramics [16,17].

However, the potential applications of ACP have so far been limited by its instability. It quickly transforms to hydroapatite (HA) in aqueous solution, although this process is strongly affected by various factors, such as pH, temperature, and presence of ions/additives in the solution. For example, the presence of Mg^2+^, CO_3_^2−^ (especially at levels of 4–6 wt.% in bone), P_2_O_7_^4−^ or F^−^ ions and biological macromolecules can effectively stabilize ACP in biological systems [18,19]. Ion substitution (Mg^2+^, CO_3_^2−^, Sr^2+^, Zn^2+^, etc.) has also been widely used to stabilize synthetic ACP [13,14,20,21]. The carbonate ion has an excellent stabilizing effect on ACP as well [22]. Inclusion of carbonate ions in ACP would not only bring it closer to the components of bone but could also improve the biological behavior such as bioactivity and solubility, making the compound ideal for biomedical applications [20,23].

There are a number of ways to synthesize ACP. High supersaturation and a neutral/alkaline environment are typically needed regardless of the other synthesis conditions [24,25]. In a synthetic route in water, ACP is precipitated from a solution containing a high concentration of calcium and the phosphoric precursor [26]. However, the purity and components of ACP synthesized from aqueous solution are difficult to control because of the possible hydrolysis of phosphate ions and the presence of foreign ions. ACP precipitated from aqueous solution also appears to have a short lifetime [25,27]. In order to avoid hydrolysis, synthetic methods in organic solvents [28], including solvothermal synthesis [7,29], have become popular in recent years. Organic solvents, i.e., alcohols, favor the formation of ACP because their lower dielectric constants than of water. This results in the ions in solution to be less solvated and consequence of decrease of the solubility and increase in supersaturation (also precipitation kinetics), which facilitates the amorphization [30,31,32]. 

Highly porous amorphous calcium carbonate (HPACC) has recently been developed by our laboratory [33]. It was obtained by drying a suspension of amorphous calcium carbonate (ACC) synthesized by dispersing calcium oxide in methanol under pressurized CO_2_ gas. This ACC suspension contained ACC nanoparticles with diameters <10 nm. The ACC suspension is stable and can be stored for several months at 0 °C before crystalline calcium carbonate is formed. In the present work, this ACC suspension was used as a source of CaCO_3_ in the synthesis of ACP. ACP can be formed from the reaction between calcium carbonate (CaCO_3_) and phosphoric acid (H_3_PO_4_) because of the relatively weak acidity of carbonic acid (p*K*_a1_(H_2_CO_3_) = 3.6) compared with phosphoric acid (p*K*_a1_(H_3_PO_4_) = 2.14) [34,35]. The carbonate content in the obtained ACP can be tailored by adjusting the ratio of CaCO_3_ to H_3_PO_4_ in the reaction mixture. On the other hand, the low cost of CaCO_3_ and H_3_PO_4_, and the room temperature synthesis facilitate the large-scale production of ACP materials.

We present a series of highly porous ACP compounds synthesized by introducing phosphoric acid into this ACC suspension in methanol at room temperature. The physico-chemical properties of the obtained ACPs were comprehensively characterized using a range of advanced techniques. Their stability in air condition, in an aqueous environment and in a cell culture medium was investigated as well. Mouse pre-osteoblastic cells were used to confirm the cytocompatibility of the synthesized ACPs. Finally, an anti-osteoporosis bisphosphonate drug—alendronate (AL)—was loaded into an ACP sample and the in vitro release of AL was monitored over time. 

## 2. Materials and Methods

### 2.1. Materials

Calcium oxide (CaO, Reagent grade) was purchased from Alfa-Aesar (Kandel, Germany). Methanol (>99.8%) was purchased from VWR (Radnor, PA, United States). CO_2_ (>99.998%) was purchased from Air Liquide AB (Paris, France). Phosphoric acid (PA, ≥99.999%), alendronate sodium (Pharmaceutical Secondary Standard), N-(2-Hydroxyethyl)piperazine-N′-(2-ethanesulfonic acid) (HEPES) aqueous solution (1 M, pH 7.0–7.6, sterile-filtered, BioReagent), dimethyl sulfoxide (DMSO) (sterile-filtered BioReagent ≥ 99.7%), and the live/dead double staining kit were purchased from Sigma-Aldrich Sweden AB (Stockholm, Sweden). Gibco Dulbecco’s phosphate-buffer saline (PBS), Gibco’s Alpha Minimum Essential Medium with nucleosides without ascorbic acid (α-MEM), Invitrogen presto blue cell viability reagent and Gibco TrypLE Express enzyme were purchased from Thermo Fisher Scientific (Waltham, MA, USA). All chemicals were used without further purification. 

### 2.2. Synthesis of ACPs and CaPs

The ACP samples were synthesized by introducing varying amounts of phosphoric acid into the ACC suspension described in our previous study [33]. In a typical synthesis, 2.5 g of calcium oxide was added to 150 mL methanol at 50 °C under constant stirring in a glass reaction vessel (Andrew Glass Co Ltd., Vineland, NJ, USA). When the mixture appeared homogeneous, 4 bar CO_2_ was fed into the reaction vessel and the vessel was sealed. The mixture was left stirring at 50 °C for 4 h. Thereafter, the pressure was released from the reaction vessel and the reaction mixture was centrifuged at 1357× *g* for 15 min to remove the unreacted calcium oxide. A suspension containing highly dispersed ACC nanoparticles of <10 nm diameter (detailed in our previous study) [33] was obtained after centrifugation. This ACC suspension was used to prepare the ACP samples.

Phosphoric acid was first dissolved in methanol at a concentration of 58 mg/mL. A series of phosphoric acid-methanol solutions were added to 30 mL ACC suspension (~25 mg ACC nanoparticles/mL) dropwise under vigorously stirring. The amount of phosphoric acid added ranged from 0 g, 0.32 g, 0.53 g, 0.58 g, 0.68 g, 0.78 g to 0.88 g per 30 mL ACC suspension. The obtained samples were named as ACC, ACP032, ACP053, CaP058, CaP068, CaP078 and CaP088, respectively. The prefix ACP refers to samples that were amorphous and CaP refers to samples that were crystalline (crystallinity is further discussed later). Porous ACC was acquired without adding phosphoric acid following the same method above. After stirring for 1 h, the mixture was dried at 150 °C in a ventilated oven in order to evaporate the solvent. A translucent or white powder was obtained after solvent evaporation. 

### 2.3. Characterization

Powder X-ray diffraction (PXRD) patterns were recorded using a Bruker D8 advance XRD Twin-Twin instrument (Bruker, Bremen, Germany) with Cu-Kα radiation (*λ* = 0.15418 nm), a step size of 0.04° and 2 s per step in the 2*θ* range from 10 to 70°. The Infrared (IR) spectra were recorded using a Bruker Tensor 27 spectrometer (Bruker, Bremen, Germany) coupled with a platinum attenuated total reflection diamond sample stage. Raman spectroscopy experiments were carried out using a Renishaw inVia Qontor (Wotton-under-Edge, UK) Raman microscope. The sample was placed on a glass slide and exposed to frequency-doubled Nd:YAG lasers (wavelength 532 nm) during data collection. Thermogravimetric analysis-differential scanning calorimetry (TGA-DSC) measurements were carried out using a Mettler Toledo TGA2 instrument (Schwerzenbach, Switzerland) in air with a flow rate of 40 mL/min from room temperature to 900 °C at a heating rate of 10 °C/min. Inductively coupled plasma–optical emission spectroscopy (ICP-OES) measurements were conducted using a Vista-MPX CCD Simultaneous ICP-OES instrument (Varian Inc., Palo Alto, CA, USA). X-ray photoelectron spectroscopy (XPS) experiments were conducted on a PHI Quantera II scanning XPS microprobe (Chanhassen, NM, USA). A full spectrum along with energy-resolved spectra for C 1s, O 1s and Ca 2p or P 2p. The spectra were calibrated against the C 1s peak at 284.8 eV for adventitious carbon. Scanning electron microscope (SEM) images were recorded using a Zeiss LEO 1530 scanning electron microscope (Oberkochen, Germany) operating at 2 kV. The samples were coated with a layer of gold-palladium to avoid the charging effect. Transmission electron microscope (TEM) images were acquired by a 200 kV JEOL JEM-2100F (JEOL Ltd., Akishima, Japan) equipped with a Schottky-type field emission gun and a bottom mounted Gatan Ultrascan camera (Gatan Inc., Pleasanton, CA, USA). Bright field (BF)- and high-angle annular dark-field (HAADF)-scanning TEM (STEM) images were simultaneously recorded by a Gatan BF detector and JEOL ADF detector, respectively. The samples were dispersed onto Cu TEM grids with carbon supporting films and dried in air. Large angle X-ray scattering (LAXS) experiments were carried out using a custom-made large-angle θ−θ goniometer with MoKα radiation (*λ* = 0.71073 Å), detailed elsewhere [33]. N_2_ adsorption measurements were carried out in a Micromeritics ASAP 2020 (Norcross, GA, USA) volumetric gas adsorption analyzer. Prior to the adsorption measurements, the sample was degassed at 100 °C for 6 h under dynamic vacuum (1 × 10^−4^ Pa) using a Micromeritics Smart VacPrep060 (Norcross, GA, USA) sample preparation unit. The procedures for the cell study, drug loading and in vitro drug release are detailed in the Supporting Information.

## 3. Results and Discussion

### 3.1. Synthesis and Characterization of ACPs and CaPs

We synthesized ACPs by introducing from 0.32 to 0.88 g of phosphoric acid to a 30 mL ACC suspension in methanol (with an estimated solid ACC content of ~25 g/mL). ACP nanoparticles in a suspension in methanol were subsequently obtained based on the reaction between the ACC nanoparticles and phosphoric acid. ACP nanoparticles aggregated randomly to form porous ACP when the solvent (methanol) was evaporated through a drying process. The obtained ACPs were characterized by various characterization techniques. 

**Figure 1:**
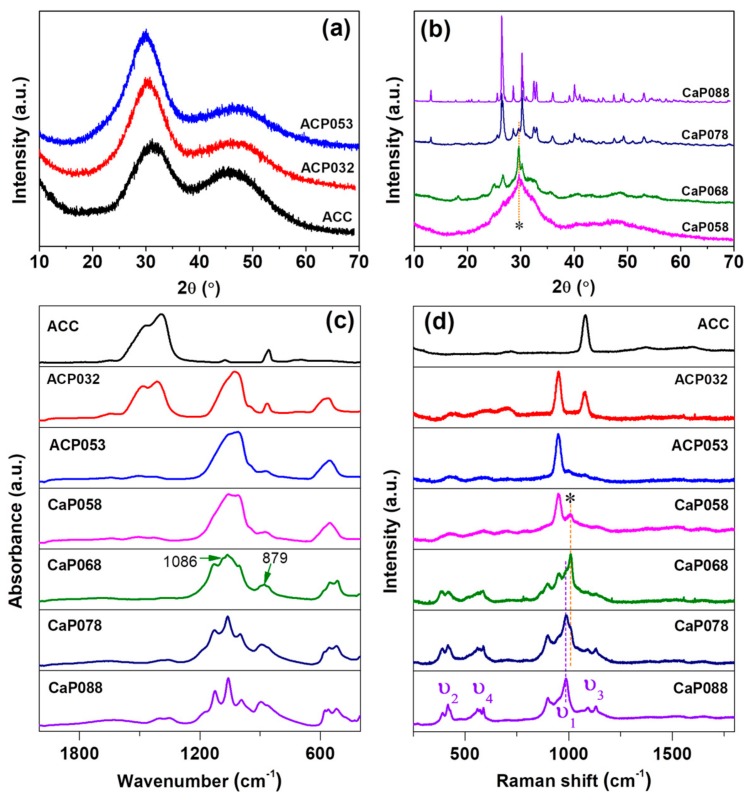
Powder X-ray diffraction (PXRD) for calcium phosphate formed by the addition of increasing amounts of phosphoric acid to amorphous calcium carbonate (ACC) suspension. (**a**) amorphous calcium phosphate (ACP) samples formed with low phosphoric acid amounts and (**b**) crystalline CaP samples formed with higher amounts; (**c**) Infrared (IR) spectra and (**d**) Raman spectra for ACP and crystalline CaP samples compared with ACC.

PXRD patterns (Figure 1a) showed that ACC, ACP032, and ACP053 (synthesized with relatively low amount of phosphoric acid) had no diffraction peaks in their X-ray diffractograms. We therefore considered these samples as X-ray amorphous (simply referred to as “amorphous” in the discussion). When the amount of phosphoric acid was further increased (from 0.58 to 0.68 g/30 mL ACC suspension), X-ray diffraction peaks related to calcium hydrogen phosphate (CaHPO_4_) were observed. Here, we used “CaP” to indicate the crystalline state of the samples (i.e., CaP058, CaP068). On these samples, calcite was also detected (the diffraction peaks of calcite are marked with an asterisk (*) in Figure 1b at around 2*θ* = 29.6 °). The formation of calcite was probably related to the formation of water as a product of the reaction between calcium carbonate and phosphoric acid—since water can induce the crystallization of ACC to calcite. When the amount of phosphoric acid was further increased (CaP078, CaP088), the diffraction peaks of CaHPO_4_ increased in intensity. The crystallinity of the CaP samples (CaP058, CaP068, CaP078 and CaP088) increased with increasing amounts of phosphoric acid added to the ACC suspension. On the other hand, the diffraction peaks related to calcite decreased in intensity with an increasing amount of phosphoric acid. The crystalline CaHPO_4_ phase was verified to be mainly monetite (PDF 00-009-0080) (Figure 1b and Figure S1). 

IR spectra of ACC, and the ACPs and CaPs are presented in Figure 1c. ACP032 displayed the characteristic IR bands of ACC (*ν*_3_ bands at 1414 cm^−1^ and 1484 cm^−1^, *ν*_1_ band at 1074 cm^−1^) as well as broad bands associated with ACP (*ν*_3_ at 1028 cm^−1^ with a shoulder at ~1072 cm^−1^, *ν*_4_ centered at ~570 cm^−1^) [25]. The broad IR bands confirmed that ACP existed in an amorphous state in ACP032, as crystalline CaP would show relatively sharp IR bands. The IR bands associated with ACC were not detected for ACP053, but the characteristic broad-bands associated with ACP were still present. When the amount of phosphoric acid was increased further, the IR bands associated with crystalline CaP (CaHPO_4_, monetite) became increasingly defined, as observed in the IR spectra for CaP058, CaP068, CaP078, and CaP088. Several sharp *ν*_4_ bands were noted between 480 and 590 cm^−1^ as well as two sharp *ν*_3_ bands between 1055 and 1125 cm^−1^. The *ν*_1_ vibration band at around 995 cm^−1^ also appeared in the CaP068, CaP078, and CaP088 spectra. The very weak band at 1086 cm^−1^ and 879 cm^−1^ in the spectrum of CaP068 may be associated with the *ν*_1_ and *ν*_3_ bands of calcite. No IR bands associated with ACC/calcite were clearly detected in the CaP058, CaP078, and CaP088 spectra. 

The Raman spectra of ACC, and of the ACP and CaP samples are shown in Figure 1d. The Raman spectrum of ACP032 had two distinct bands: one at ~1100 cm^−1^, which corresponded to the *ν*_1_ vibration of the carbonate ion (CO_3_^2−^), and one at ~950 cm^−1^, which corresponded to the *ν*_1_ vibration of the phosphate ion (PO_4_^3−^). In the Raman spectrum of ACP032, other vibration bands (*ν*_2_, *ν*_3_ and *ν*_4_) typically observed in crystalline CaP had very low intensities and could not be easily identified. The broad *ν*_1_ and lack of the crystalline CaP Raman bands further confirmed the amorphous state of ACP032. The Raman spectrum of ACP053 had similar bands, associated with PO_4_^3−^, to those seen in ACP032. However, the *ν*_1_ band of CO_3_^2−^ was not observed because of the reduced carbonate content. For CaP068, CaP078, and CaP088, the Raman bands associated with crystalline CaP (CaHPO_4_, monetite) were distinct and clearly observed. In addition to the *ν*_1_ band at around ~987 cm^−1^, the *ν*_2_ (~380–430 cm^−1^), *ν*_3_ (at around ~1092 and 1129 cm^−1^), and *ν*_4_ (~540–610 cm^−1^) bands were clearly observed for these samples [36,37,38]. The Raman bands marked with asterisk (*) could be attributed to the small amount of calcite in CaP058, CaP068 and CaP078, which was coincident with the PXRD results (Figure 1b). 

**Figure 2:**
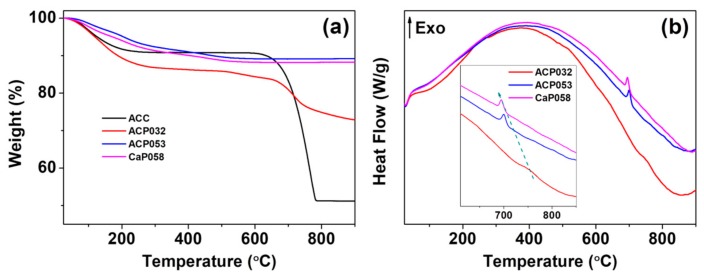
(**a**) Thermogravimetric analysis (TGA) and (**b**) the corresponding differential scanning calorimetry (DSC) heat-flow curves for the amorphous calcium phosphate samples ACP032 and ACP053 and the crystalline calcium phosphate sample CaP058.

We further analyzed the ACPs and CaPs using TGA-DSC. The TGA curves for ACP032, ACP053, and CaP058 are shown in Figure 2a (TGA-DSC curves for the other CaPs can be found in Figure S2 in the Supporting Information). The TGA curves of ACP032 indicated two obvious mass losses. The first was related to the evaporation of adsorbed species and structural water below ~500 °C. The second that occurred at ~750 °C (Figure 2a) was related to the decomposition of crystalline calcium carbonate (formed by heating ACC) in ACP032. The carbonate content of ACP032 was calculated to be around 15 wt.% (Figure S3) according to the mass drop between 600 °C and 800 °C in the TGA curve of ACP032. The presence of carbonate in as-synthesized ACP032 was further investigated by ICP-OES (Table 1) and XPS (Figure S4). The composition of ACP032 from ICP-OES suggested that it contained about 16 wt.% carbonate, which was coincident with the value calculated from TGA. The TGA curves for ACP053 and CaP058 showed a gradual decrease in mass up to ~500 °C; this mass loss was related to the removal of adsorbed and structural water (and condensation reaction of CaHPO_4_ in CaP058, which was also observed in CaP068, CaP078, and CaP088, as discussed later) [39]. No mass drop related to the decomposition of calcium carbonate was observed for ACP053 and CaP058. The data from ICP-OES (Table 1) suggested that ACP053 contained a small amount (~4 wt.%) of carbonate. The presence of a small amount of carbonate is typical for ACP and CaP materials and has been reported previously in literature [28,36,40,41,42]. It is worth noting that the content of carbonate ions in ACP053 was similar to that in human bone [43]. The different carbonate content in ACP032 and ACP053 also suggests that the carbonate constitution in ACP materials could be easily adjusted with this method. A small exothermal peak at about 700 °C, related to the phase transformation/crystallization of ACP, was observed in the DSC heat-flow curves of ACP053 and CaP058 (Figure 2b) [44,45]. This exothermic peak shifted from 700 °C in ACP053 to 694 °C in CaP058. This shift may be related to the low level of crystallinity present in the as-synthesized CaP058 (Figure 1b). The presence of small crystallites may have favored the further crystallization of ACP to CaP. 

The TGA curves of CaP068, CaP078, and CaP088 (Figure S2) showed that, apart from evaporation of adsorbed or structure water, there was a clear mass loss between 300 and 500 °C; the magnitude of the mass loss increased with increased crystallinity in the CaP samples. This mass loss was attributed to the condensation reaction of monetite (i.e., CaHPO_4_) [39]. Differences in the TGA-DSC analysis of CaP068, CaP078 and CaP088 are presented and discussed further in Figure S2 in the Supporting Information. 

**Figure 3:**
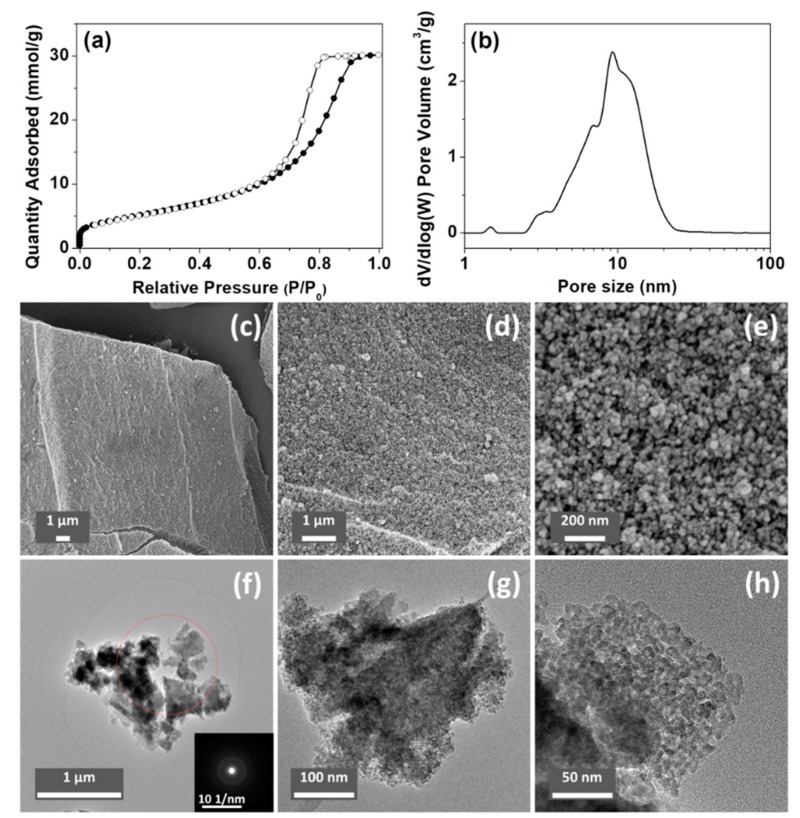
(**a**) N_2_ sorption isotherm and (**b**) density functional theory (DFT) pore-size distribution curve of ACP053. (**c**–**e**) scanning electron microscope (SEM) images and (**f**–**h**) transmission electron microscope (TEM) images of ACP053 (the insert in Figure 3f is the corresponding selected-area electron diffraction pattern).

The porosity of the ACPs and CaPs was analyzed using N_2_ sorption; the results are summarized in Table 2. The Brunauer–Emmett–Teller (BET) surface areas of ACP032 and ACP053 (the synthesis of which was based on the synthesis of HPACC [33]) were higher than those of all the other tested samples, and higher than that of HPACC in our previous study. Specifically, ACP053 had the highest BET surface area (418 m^2^/g) of all the forms of CaP (both amorphous and crystalline) reported in the literature [7,28,46,47,48], to the best of our knowledge (as ACP032 contained around 16 wt.% of carbonate, it was not considered a high purity ACP). A comparison between the porosity of the ACPs synthesized in this work and that of the ACPs reported in the literature is listed in Table S1. The N_2_ adsorption/desorption isotherm and the density functional theory (DFT) pore-size distribution for ACP053 are shown in Figure 3a,b (N_2_ sorption isotherms and the DFT pore-size distribution curves for other samples can be found in the Supporting Information, Figure S5). 

The SEM and TEM images of ACP053 are shown in Figure 3c–h. ACP053 had similar nanostructure as HPACC, which was constructed with aggregated nanoparticles. The ACP053 nanoparticles had no well-defined shape (e.g., exact spherical particles) or size according to the images shown in Figure 3c–h. From the high magnification images (Figure 3e,h), it can be seen that some of the ACP053 nanoparticles showed a certain level of coalescence. The nanoparticles on ACP053 appeared to be less uniformly shaped than those in HPACC. This was probably the reason behind the broader pore size distribution observed on ACP053 when compared with HPACC. These nanometer-sized ACP particles (<10 nm) appeared to have aggregated together without any particular order, similar to that observed in previous studies [28]. The porosity of ACP053 is the result of the space between these nanometer-size particles. The irregular porous structure of ACP053 was further confirmed by the STEM images shown in Figure S6. The average diameter of the individual particles was estimated, using the BET surface area and the density of ACP053 (2.59 g/cm^3^, obtained by He pycnometry), to be ~5.6 nm (modeled with non-porous spherical particles), which was in agreement with the TEM image shown in Figure 3h. The selected-area electron diffraction pattern in the insert of Figure 3f further confirmed the amorphous state of ACP053, even on a nanometer scale. The structure of the ACP032 particles was similar to those of ACP053 according to the SEM, TEM, and STEM images shown in Figures S7 and S8. In contrast, the crystalline CaP088 particles had plate-like structure with diameters of several micrometers, as shown in the SEM images in Figure S9. 

In the rest of this study, we will focus only on the ACP032 and ACP053. These samples had high BET surface area that allow them to be utilized as potential functional materials for applications such as drug delivery and biomedicine. 

**Figure 4:**
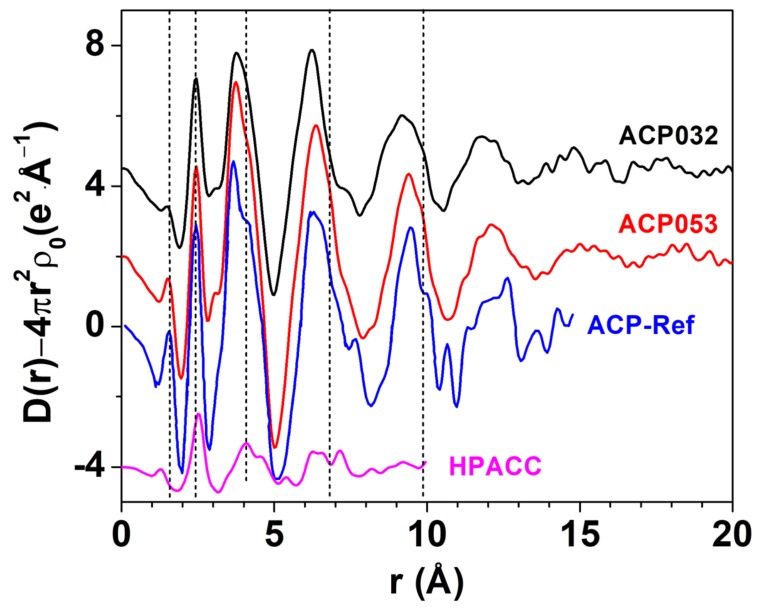
The large angle X-ray scattering (LAXS)-radial distribution function (RDF) of ACP053, ACP032, ACP-Ref and highly porous amorphous calcium carbonate (HPACC). The curves have been shifted vertically for better visibility. The ACP-Ref data were sourced and rescaled from Ref. [25], while the HPACC data were obtained by us and presented in a previous work Ref. [33].

LAXS was employed to determine the structure of ACP053. The LAXS radial distribution function (RDF) of ACP053 is shown in Figure 4 together with those of APC032 and HPACC [33]. The RDF for ACP published by Ranz et al. [49] (labeled as ACP-Ref in Figure 4) is included for comparison. The structures of the ACP053 and ACP032 samples were very similar to that of ACP-Ref, indicating that the calcium phosphate structure dominated during particle formation. The first peak in ACP-Ref at around 1.54 Å corresponded to the P-O distance in PO_4_^3−^ or HPO_4_^2−^ ions [50,51]. The first peak in the HPACC sample corresponded to the C-O distance at ca. 1.29 Å [52]. For ACP053, the position of the first peak was identical to that reported for ACP-Ref, but, in ACP032, it was at a slightly shorter distance in accordance with the chemical composition of the sample. This suggested that ACP032 resembles both ACP-Ref and HPACC. The C-O peak in ACP032 was not clearly visible, which could be attributed to the low carbonate content in ACP032 (~16 wt.%) and less pronounced C-O peak when compared with the P-O peak. The peak at around 2.39 Å in the RDFs for ACP053 and ACP032 was associated with the Ca-O distance in the 6-coordination, octahedral geometry of oxygens of PO_4_^3−^ or HPO_4_^2−^ ions around the Ca^2+^ ions, similar to that observed for ACP-Ref. For HPACC, the corresponding peak was observed at a significantly longer distance (2.52 Å). This is due to 8-coordinated Ca^2+^ in HPACC as discussed in our previous work [33]. The Ca-O distance in ACP053 and ACP032 appeared to be more similar to that in ACP-Ref than that in HPACC, which further corroborated that the ACP structure type dominated in ACP032 and ACP053 regardless of the amount of carbonate ions present. However, the shoulders in the RDF at about 4.05 Å, 6.80 Å and 9.85 Å (short dashed lines in Figure 4), which are fingerprinting features for ACP, appeared more pronounced in the RDF of ACP053. Both ACP053 and ACP032 showed distinct peaks up to 17.5 Å suggesting that, although these two samples were amorphous, they had a detectable short-range atomic order. The short-range atomic order observed for ACP053 was much more pronounced than the reported short-range atomic order up to 9.5 Å for ACP (of which the basic structural unit is the so-called Posner’s cluster) with a formula of Ca_9_(PO_4_)_6_ [24,25,50]. The fact that the RDFs for ACP053, ACP032, and ACP-Ref were in principle superimposable implies that ACP053 and ACP032 had similar building blocks to ACP-Ref. 

### 3.2. Stability of ACPs

The stability of the synthesized ACPs was tested under different storage conditions. When stored in open air (relative humidity of approximately 43%), ACP053 and ACP032 remained amorphous even after 14 months as confirmed by PXRD and IR (Figure S10). The storage stability observed here appeared to surpass that reported for other ACPs in the literature (Table S2). The excellent stability of the ACPs may have been attributed to the presence of CO_3_^2−^, as revealed by ICP-OES (Table S1), and the unique synthesis process (e.g., synthesized in methanol and dried at 150 °C). The long-term storage stability in an amorphous state in air conditions exhibited by ACPs is a key advantage for their potential use in various applications. The decrease in the BET surface area of ACP053 was ~60% after 12 months (Figure S11). This decrease in BET surface area was probably related to intergrowth of the nanometer-sized particles. The increased pore size of ACP053 after storage for 12 months in open air also suggests that the drop in BET surface area was related to the growth of nanoparticles. The same observation has been noted for other similarly structured materials, such as mesoporous magnesium carbonate and HPACC [33,53]; their stability was increased by loading them with guest molecules such as additives or pharmaceuticals. 

**Figure 5:**
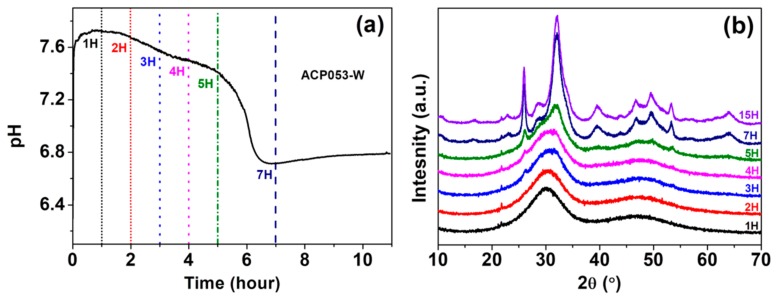
(**a**) pH changes over 1–7 h in a dispersion of ACP053 in de-ionized water and (**b**) powder X-ray diffraction (PXRD) patterns for ACP053 exposed to de-ionized water for 1–15 h.

We have also studied the stability of ACP053 in de-ionized water. Crystallization of ACP in water is typically accompanied by an abrupt drop in pH [21,54]. The pH change of a dispersion of 0.1 g of ACP053 in 10 mL de-ionized water at room temperature was followed in order to monitor the crystallization process; Figure 5a shows the pH of ACP053 dispersed in de-ionized water over time. The initial pH of the de-ionized water was ~6.8, rising to 7.7 in the first 30 min after adding ACP053. This rise in pH is presumably due to the high solubility of ACP053 and the small amount of carbonate present. The pH remained stable during the first 2 h followed by a gradual decrease over the next 3 h. The pH was about 7.4 five hours after the addition of ACP053. During the following two h, there was a significant drop in the pH of the dispersion (to ~6.7). The PXRD patterns for ACP053 dispersed in de-ionized water over the same time frame are shown in Figure 5b. According to these PXRD patterns, ACP053 remained amorphous for up to 3 h in water. Thereafter, crystallization was noted by the appearance of a weak diffraction peak at 2*θ* = 26°. The intensity of this peak increased further after 5 h of exposure to water. After 7 h, ACP053 had been converted to HA (Figure S12a). The time-resolved PXRD patterns shown in Figure 7b were in agreement with the results obtained in the pH study as described above. In summary, ACP053 was generally stable in water and remained amorphous for the first 2 h. Thereafter, a phase transition from ACP053 to HA did take place with increasing rate with time, especially after five hours. These results are comparable with ion-doped ACPs reported in the literature [21]. After 7 h of exposure to water, the PXRD pattern (Figure 5b and Figure S12a) showed that HA had formed. The time-resolved IR spectra (Figure S12b) also confirmed that ACP053 was stable during the first 2 h but had fully crystallized after 7 h. The broad *ν*_3_ vibration band of PO_4_^3−^ at 1050 cm^−1^ became a sharp band at 1025 cm^−1^ with a shoulder at around 1100 cm^−3^ and the *ν*_4_ band at around 560 cm^−1^ had split into two bands at 602 and 561 cm^−1^. These new vibration bands are characteristic for HA [40]. The SEM images of ACP053 after 15 h of exposure to water are shown in Figure S13. These images showed that the nanometer-sized particles observed in Figure 5 had aggregated and crystallized into crystals of around 100–200 nm in the longest dimension. The general morphology of the HA formed from ACP053 after exposure to water for 15 h was similar to that of carbonated HA crystals found in bones (with a length of about 100 nm, width of 20–30 nm, and thickness of 3–6 nm) [55]. It has been documented that the nanometer-sized HA crystals exhibit biological properties such as non-toxicity, and higher bio-resorbability and osteoblast adhesion compared with crystalline CaP [10,56,57]. The formation of HA crystallized from ACP053 could, therefore, be a promising candidate for bone regeneration applications. ACP032 was also tested for its stability when exposed to de-ionized water. It had an extended lifetime (>20 h) in de-ionized water without any crystallization (see Supporting Information, Figures S14 and S15), surpassing the data reported for other ACPs [21,26,58]. The SEM images of ACP032 exposed to water for 50 days (Figure S16) showed relatively large calcite crystals and small HA crystals. This observation fitted well with the PXRD patterns (Figure S15) where strong diffraction peaks for calcite and weak diffraction peaks for HA were observed independently. These calcite and HA crystals formed separately from each other (i.e., no particle intergrowth), which is in agreement with the observation made by Kababya et al. on phosphate-doped ACC [59]. During the crystallization process, the formation of calcite was faster than that of HA and dominated in the overall transformation, as seen from the time-resolved IR spectra (Figure S14) and PXRD patterns (Figure S15). The stability study carried out here further confirmed that the carbonate content in ACP played a significant role in its stability and in the formation of HA from ACP [60]. A comparison of the stability of different ACPs in water is summarized in Table S3. 

### 3.3. Cytocompatibility Study of ACPs

**Figure 6:**
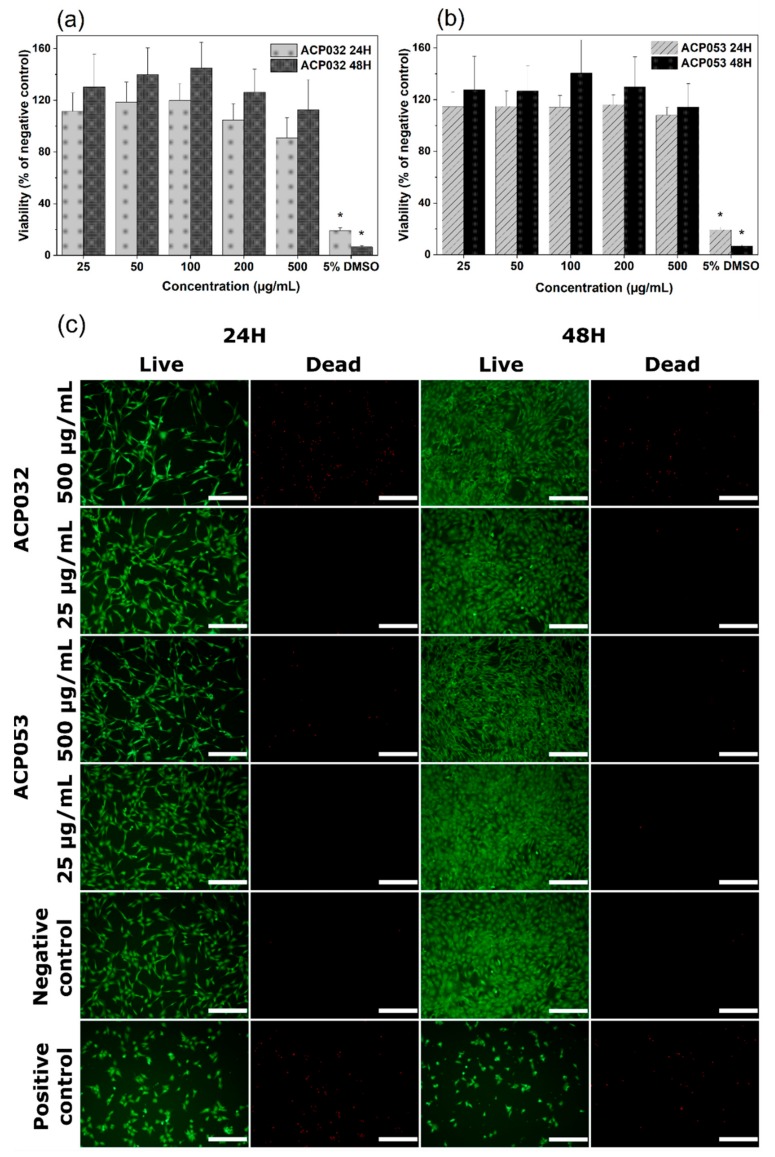
Cell viability of MC3T3 cells exposed to varying concentrations of (**a**) ACP032 and (**b**) ACP053 for 24 h and 48 h. The data are presented as means ± standard error of the mean for *n* = 6, where significant differences (*p* < 0.05) compared to the negative control are marked with an asterisk (*); (**c**) live/dead staining of MC3T3 cells exposed to 500 µg/mL or 25 µg/mL of the amorphous calcium phosphate samples ACP032 and ACP053 for 24 h and 48 h. The negative and positive controls were unexposed cells and cells treated with 5% DMSO, respectively. The scale bar in Figure 6c is 100 µm.

The cytocompatibility of calcium carbonate and calcium phosphates with osteoblastic cells has been well documented over the years [1,2,3,61,62,63,64,65]. However, changes in the physico-chemical properties of the material, such as crystallinity, porosity, and particle size and morphology, can have notable effects on the response of exposed cells [66]. Therefore, mouse pre-osteoblastic cells (MC3T3) were chosen to conduct an in vitro cytotoxicity study as a first step in the biocompatibility evaluation of the newly synthesized ACPs, bearing in mind the potential use of the composites for bone regeneration and drug delivery applications. The metabolic activity of cells treated with ACP032 and ACP053 at concentrations from 25 µg/mL to 500 µg/mL for 24 h and 48 h did not significantly differ from that of untreated cells (the negative control) (Figure 6a,b). The percentage of cell viability respect to the negative control is above the 70% toxicity limit established by the International Organization for Standardization (ISO) standard (ISO 10993-5:2009) [67], for all sample concentrations and both exposure times. 

Live/dead staining of MC3T3 cells was employed to visually evaluate any changes in the cell morphology as well as an indicator of cell membrane integrity. Two concentrations of ACP032 and ACP053, 500 µg/mL and 25 µg/mL were used for the assay. As seen in Figure 6c, the morphology and density of the viable cells after 24 h and 48 h of exposure at both high and low concentrations were comparable to those of the untreated cells (negative control). It should be noted that a relative high number of non-viable cells was observed after exposure to 500 µg/mL ACP032 (Figure 6c). However, the viable cell morphology remained fibroblast-like throughout the incubation period, in conjunction with a statistically significant (*p* < 0.05) cell proliferation after 48 h. Overall, the results indicated a non-toxic effect of the ACP materials when MC3T3 cells were exposed to the materials under the conditions of the study.

The stability of the ACPs when exposed to cell culture medium was also investigated by monitoring the PXRD pattern and IR spectra for ACP in the cell culture medium over time. Both ACP053 and ACP032 demonstrated higher stability in the cell culture medium than in de-ionized water (Figures S17 and S18). ACP053 started to crystallize to HA after 24 h, while ACP032 remained amorphous for up to 48 h. The enhanced stability in cell culture medium compared with that in de-ionized water could be related to the proteins present in the cell culture medium which may act as a stabilizer for ACPs [68]. In addition, the crystallization of ACP053 to HA over the time period of cell exposure did not seem to significantly affect the observed cell response.

### 3.4. Drug Loading and In Vitro Release of AL with ACP053 as Carrier

ACP053 was selected for further studies as a possible carrier for anti-osteoporosis bisphosphonate (BP) drugs. AL was chosen as the model drug to load into ACP053 by a soaking method as described in the experimental section. The loading percentage of AL was determined to be ~5.6 wt.% and loading efficiency was ~87.88%, which is comparable to the value reported in literature [69]. The ACP053 loaded with AL (ACP053-AL) remained amorphous, as seen in the PXRD pattern (Figure S19). ACP053-AL had a BET surface area of ~370 m^2^/g and a pore volume of 1.11 cm^3^/g (N_2_ sorption isotherms in Figure S20). The high porosity of ACP053-AL implied that AL was loaded into the pores of ACP053. The pore volume of ACP053-AL was 1.11 cm^3^/g, comparable to the expected value calculated based on full AL adsorption on the pore surface (i.e., not on the surface of the ACP053 particles) of 1.07 cm^3^/g. The high pore volume on ACP053-AL was a clear indication that AL was not adsorbed on the surface of ACP053 and not blocking the pore opening of ACP053.

**Figure 7:**
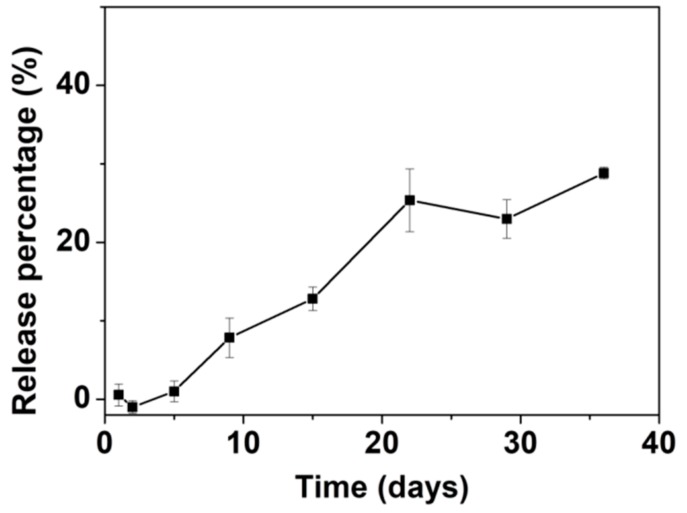
Graph showing the release of alendronate (AL) from ACP053-AL in N-(2-Hydroxyethyl)piperazine-N′-(2-ethanesulfonic acid) (HEPES) buffer (10 mM, pH = 7.0–7.6). All measurements were performed in triplicate and the mean concentrations and corresponding deviations were calculated.

The in vitro release of AL was assessed in an HEPES buffer saline (10 mM, pH = 7.0–7.6). The AL release curve is shown in Figure 7. Up to ~25% of the total amount of loaded AL was released in 22 days. After 22 days, the release of AL appeared to fluctuate at around the same level and no further significant release of AL was noted up to 40 days. The slow release rate and limited release percentage of AL are comparable to those of other materials tested for loading and release of AL [70,71] as well as other BPs [55,72,73]. Our group previously reported loading of the BP pamidronate within biomimetic HA with a non-measurable release [74]. Palazzo et al. reported the loading and release of AL with biomimetic HA as the carrier; the release percentage was ~20% after one day with a slight increase to ~25% after 18 days [55]. Kim et al. loaded AL into CaP microspheres in situ, with a loading percentage of ~14.4 wt.% [73]. The AL-loaded CaP released ~18% of the AL after 20 days followed by continuous release of up to ~24% after 40 days. The slow release rate and limited release percentage may be related to the strong interaction between AL and ACP053. It has been documented that there are significant differences in the affinity constants for HA among the BPs, with a rank order of zoledronate > alendronate > ibandronate > risedronate > etidronate > clodronate [75]. Palazzo et al. proposed that the adsorption of negatively charged AL is favored on the calcium-rich HA surface [55]. As for ACP053, the high specific surface area and the presence of a small amount of carbonate (~4 wt.%) could enhance the adsorption/loading of AL into ACP053. This could result in the slow release rate in vitro as observed. On the other hand, the limited release percentage of AL demonstrated by ACP053 may not necessarily be a drawback for some applications. The incomplete release of AL from ACP053 could be beneficial for the formation of dense bone structures in, for example, bone healing application. The dissolution of ACP053 is retarded by the presence of AL and the AL could also inhibit the formation of osteoclast. The lack of osteoclast would allow the osteoblasts to form a dense bone structure [74]. 

## 4. Conclusions

A series of ACPs and crystalline CaPs were successfully synthesized by introducing phosphoric acid into an ACC suspension in methanol at room temperature. ACPs with varying levels of carbonate content (from 4–16 wt.%) showed increased BET surface areas (over 400 m^2^/g) over other ACPs previously reported in literature. The synthesized ACPs were composed of small ACP nanoparticles of several nanometers in size. These ACP nanoparticles had a short-range atomic order up to 20 Å and were stable and remained amorphous for more than one year in air. When exposed to de-ionized water, the ACPs remained amorphous for a number of hours, depending on the carbonate content, but converted to HA with time. The ACPs were cytocompatible with bone cells in vitro and ACP053 had the ability to carry and release bisphosphate (e.g., AL). As ACP is a precursor for the formation of the important biomaterial HA, the ability to obtain ACP with high porosity and long-term storage stability is promising for the development of ACP for certain bio-medical applications, such as bone therapy and drug delivery. For example, the HA formed from ACP053 exposed in de-ionized water exhibited similar microstructure as carbonated HA crystals found in bones, which indicated the potential applications of this HA in bone healing. This ACP material could be used a gradient for calcium phosphate-based cement as its high bioactive. The high porosity would facilitate the high loading content and pH sensitive property of ACP could also endow the pH sensitive release of drugs. The performance of these high surface area and stable ACPs in these potential applications should be further explored. 

## Figures and Tables

**Table 1:** Inductively coupled plasma–optical emission spectroscopy (ICP–OES) analysis of the element content in the amorphous calcium phosphate samples ACP053 and ACP032.

	C	H	Ca	P	Ca/P	Estimated Carbonate Content (wt.%)
**ACP032**	0.26	1.56	0.84	0.38	**2.21**	**~16%**
**ACP053**	0.07	1.73	0.86	0.56	**1.53**	**~4%**

**Table 2:** Porosity of ACP and crystalline CaP samples synthesized in this work.

Samples	Brunauer–Emmett–Teller (BET) Surface Area (m^2^/g)	Peak Pore-Size ^a^ (nm)	Pore Volume ^b^ (cm^3^/g)	Density (g/cm^3^) ^c^	Calculated Particle Size ^d^ (nm)
ACP032	423	5.4	0.57	2.49	5.7
ACP053	418	9.3	1.05	2.59	5.6
CaP058	294	18.6	1.39	2.61	7.8
CaP068	133	18.6	0.62	2.70	16.7
CaP078	52	6.8	0.11	-	-
CaP088	6	18.6	0.10	-	-

(**a**) peak pore-size distribution was calculated by using the density functional theory (DFT) to the adsorption points using the N_2_ slit pore model; (**b**) pore volume was taken at the last adsorption point at relative pressure ~0.98; (**c**) density of samples was obtained by He pycnometry; (**d**) particle size was calculated by Brunauer–Emmett–Tell (BET) surface area and density of sample (modelled with non-porous spherical particles).

## Supplementary Materials

The following are available online at https://www.mdpi.com/2079-4991/10/1/20/s1, Figure S1: PXRD pattern for CaP088; Figure S2: TGA and DSC curves for CaP068, CaP078 and CaP088; Figure S3: TGA curve of ACP032; Figure S4: High-resolution X-ray photoelectron spectroscopy of ACP053 and ACP032; Figure S5: N_2_ adsorption-desorption isotherms and density functional theory pore-size distribution curves; Table S1: Comparison of the synthesis methods, particle sizes, BET surface areas, pore volumes, pore sizes and applications of ACP samples synthesized in this work and other ACP and crystalline CaP samples reported in the literature; Figure S6: STEM images of ACP053; Figure S7: SEM and TEM images of ACP032; Figure S8: STEM images of ACP032; Figure S9: SEM images of CaP088; Figure S10: PXRD patterns and IR spectra ACP053 and ACP032 after storage under ambient conditions for 14 months; Table S2: Comparison of the stability of ACP samples synthesized in this work or reported in the literature; Figure S11: N_2_ adsorption/desorption isotherms and density functional theory pore-size distribution curves for ACP053 as synthesized and after storage under ambient conditions for 12 months; Figure S12: PXRD pattern for ACP053 after exposure to de-ionized water for 15 h and IR spectra for ACP053 after immersion in de-ionized water for 1–15 h; Figure S13: SEM images of the hydroxyapatite formed from ACP053 after exposure to de-ionized water for 15 h; Figure S14: IR spectra for ACP032 after exposure to de-ionized water for 1 h to 50 days and IR spectra for ACP032 after exposure to de-ionized water for 1 hour and 30 days; Figure S15: PXRD patterns for ACP032 after exposure to de-ionized water for 1 h to 50 days and 30 days; Figure S16: SEM images of ACP032 after exposure to de-ionized water for 50 days; Figure S17: PXRD patterns IR spectra for ACP053 after exposure to cell culture medium for 5–48 h; Figure S18: PXRD pattern and IR spectra for ACP032 after exposure to cell culture medium for 5–48 h; Figure S19: PXRD patterns for ACP053 before and after loading with alendronate; Figure S20: N_2_ sorption isotherms for ACP053 before and after loading with alendronate and the corresponding density functional theory pore-size distribution graphs.
