# Supplementary material for: Highly Porous Amorphous Calcium Phosphate for Drug Delivery and Bio-Medical Applications
Source: Nanomaterials (Basel). 2019 Dec 19;10(1):20. doi: 10.3390/nano10010020 (PMC7022897; doi:10.3390/nano10010020)
Supplement: Supplementary file 1 [file nanomaterials-10-00020-s001.pdf]

Supplementary Materials: Highly Porous Amorphous Calcium Phosphate for Drug Delivery and Bio-Medical Applications

Rui Sun [1], Michelle Åhlén [1], Cheuk-Wai Tai [2], Éva G. Bajnóczi [3], Fenne de Kleijne [1], Natalia Ferraz [1], Ingmar Persson [3], Maria Strømme [1,*] and Ocean Cheung [1,*]

1 Division of Nanotechnology and Functional Materials, Department of Engineering Sciences, Uppsala University, SE-751 21 Uppsala, Sweden; rui.sun@angstrom.uu.se (R.S.); michelle.ahlen@angstrom.uu.se (M.Å.); fenne.dekleijne@student.fontys.nl (F.d.K.); natalia.ferraz@angstrom.uu.se (N.F.)

2 Department of Materials and Environmental Chemistry, Stockholm University, SE-106 91 Stockholm, Sweden; cheuk-wai.tai@mmk.su.se

3 Department of Molecular Sciences, Swedish University of Agricultural Sciences, SE-750 07 Uppsala, Sweden; eva.bajnoczi@slu.se (É.G.B.); ingmar.persson@slu.se (I.P.)

* Correspondence: maria.stromme@angstrom.uu.se (M.S.); ocean.cheung@angstrom.uu.se (O.C.)

S1. Experimental Section

S1.1. Large angle X-ray scattering (LAXS)

The LAXS measurements of ACP samples used the same method for HPACC as detailed in our previous work [1].

The reduced intensity function is Fourier transformed in order to get the radial distribution function (RDF) according to the following equation:

$$D(r) - 4\pi r^2 \rho_0 = \left(\frac{2r}{\pi}\right) \int_{s_{min}}^{s_{max}} s \cdot i(s) M(s) \sin(rs)\, ds$$

where $s = (4\pi \sin\theta)/\lambda$ is the scattering variable, $M(s)$ is a modification function and ρ_0 is the average scattering density.

S1.2. Cell study

Mouse pre-osteoblastic cells (MC3T3, subclone 14, CRL-2594, American Type Culture Collection (ATCC)) were cultured in alpha minimum essential medium with nucleosides and no ascorbic acid (α-MEM, Gibco) supplemented with 10 v/v% fetal bovine serum (FBS) and 1% penicillin/streptomycin (10,000 U/ml penicillin and 10,000 µg/ml streptomycin). Cells were incubated at 37˚C in 5% CO2 and a humidified atmosphere for 48 ± 2 hours and subcultured when they reached approx. 80% confluency using TrypLE Express enzyme.

For the exposure experiments, cells were seeded in clear 96-well tissue plates at a density of 3200 cells per well (200 µl) and incubated for 24 hours. The cell culture medium in the wells was removed after the initial incubation time and material dispersions of ACP032 and ACP053, at concentrations of 500 µg/mL, 200 µg/mL, 100 µg/mL, 50 µg/mL and 25 µg/mL, in cell culture medium were added to the wells. Untreated cells were the negative control and cells exposed to cell culture medium supplemented with 5 v/v% DMSO served as the positive control. Cells were further cultured for 24 ± 2 hours and 48 ± 2 hours at 37˚C, in 5% CO2 and a humidified atmosphere. The cell culture medium was removed from the wells after the incubation period and the cells were washed with PBS. 200 µL of a 10 v/v% presto blue solution in cell culture medium was added to each well and incubated for 60 min. 100 µL from each well was then transferred to black 96-well plates and the fluorescence was measured

at wavelengths of λex. = 560 nm and λem. = 590 nm using a Tecan Infinite M200 spectrofluorometer (Männedorf, Switzerland).

MC3T3 cells exposed to ACP032 and ACP053 dispersions at 25 μg/mL and 500 μg/mL of each respective material, untreated cells and cells exposed to 5% DMSO were stained with calcein-acetoxymethyl (AM) and propidium iodide (PI) and thereafter visualized by fluorescence microscopy. Briefly, after exposure for 24 ± 2 hours and 48 ± 2 hours, the cell culture medium in the wells was removed and 100 μL of a dye solution consisting of 2 μL calcein-AM and 1 μL PI per ml of cell culture medium was added per well. After incubation at 37°C in 5% CO2 and a humidified atmosphere for 15 min, the stained cells were imaged using a Nikon Eclipse TE2000 microscope (Minato, Tokyo, Japan) with a filter set at λex. = 490 nm, λem. = 515 nm for viable cells and λex. = 535 nm, λem. = 617 nm for dead cells.

Cell viability data were log-transformed and analyzed using Welch's ANOVA followed by the Games-Howell post-hoc test using R Studio software (v.3.5.2), where differences from the negative control (untreated cells) were considered statistically significant when p was < 0.05. All experiments (n = 6) were performed in triplicate.

S1.3. Drug load and in vitro release

AL was loaded into ACP by the soaking method. Specifically, 1.0 g ACP was added to 150 mL methanol solution containing AL at a concentration of 90 μg/mL. After shaking at 200 rpm on a shaker (BioSan Orbital Shaker PSU-10i, Nordic Biolabs) for 1 day, the dispersion was centrifuged at 1357 × g for 30 min. The ACP was recovered and the AL loading procedure was repeated five times. The concentration of AL in the supernatant after each loading step was recorded by colorimetry based on the reaction of the primary amino group in AL with ninhydrin in a methanol medium in the presence of 0.05 M sodium bicarbonate [2]. The color product had UV absorption at 568 nm on a UV-vis spectrophotometer (Shimadzu UV-1800 spectrophotometer with 1 cm matched cells). After carrying out the loading procedure five times, the obtained ACP-AL was dried at 70 °C in a ventilated oven for 24 hours.

The calibration curve was made from four solutions with concentration 6.2542 μg/mL, 8.3390 μg/mL, 12.5085 μg/mL and 25.0170 μg/mL. These four concentrations were in the linearity range (3.75—45 μg/mL) in the reference [2]. The acquired calibration curve was $y = 0.02396x + 0.00203$, (y is the instrument response, i.e. absorbance and x is the concentration in unit of μg/mL, R^2=0.99999). The limit of detection (LOD) was further calculated by LOD = 3.3 x σ / S (S is the slope of the calibration curve and σ is the standard deviation of the response) [3]. The LOD of the method used in this study was 0.058 μg/mL. The loading content of AL in ACP was determined by the difference in the concentration of the AL-methanol solution before and after loading process. The original concentration of AL-methanol concentration is 90 μg/mL. The concentration of the solution after loading was tested by colorimetry method as discussed previously. The drug loading efficiency, as the percentage of AL mass loaded into ACP with respect to the initial AL mass was also calculated.

The in vitro dissolution tests were carried out in HEPES aqueous buffer (10 mM, pH = 7.0-7.6) (AL has solubility in water range from 10 mg/mL to 50 mg/mL [4-6]). 210 mg of AL-loaded ACP (11.76 mg AL) was added to 120 mL of 10 mM HEPES buffer in a glass laboratory bottle. The mixture was kept shaking at 200 rpm on a shaker (BioSan Orbital Shaker PSU-10i, Nordic Biolabs) at 37 °C in an oven. Aliquots of 1.2 mL were withdrawn from each vessel at regular time intervals and centrifuged at 14000 rpm for 10 min, and then 1 mL of the aliquot was used to test the amount of AL released by the colorimetric method detailed above [2]. The remaining 0.2 mL was added to 1.0 mL of fresh HEPES buffer and was reintroduced back into the bottle. The drug release experiment was repeated three times, and all measurements were performed in triplicate. The average concentration and the corresponding standard deviations were calculated.

S2. Characterization of amorphous calcium phosphate (ACP) and crystalline calcium phosphate (CaP) samples

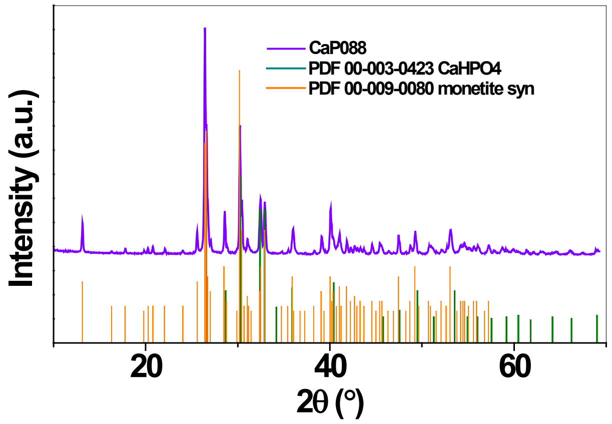

Figure S1. Powder X-ray diffraction patterns for calcium phosphate samples CaP088 fitted with the diffraction peaks of monetite (PDF 00-009-0080) and CaHPO4 (PDF 00-003-0423).

In the thermogravimetric analysis curves for CaP068, CaP078 and CaP088 (Figure S2a), the mass loss between 300 and 500 °C was attributed to the condensation reaction of monetite [7].

$$2CaHPO_4 \rightarrow Ca_2P_2O_7 + H_2O$$

The magnitude of the mass loss was in the order CaP088 > CaP078 > CaP068. This agreed well with the content of monetite inside the samples. CaP078 and CaP088 were in the crystalline form (monetite), as seen from the powder PXRD patterns in Figure 1, and therefore there were no exothermic peaks in the DSC curves, only endothermal peaks between 300 and 410 °C representing the condensation of monetite. Note that there was no indication of mass loss related to the decomposition of calcium carbonate in the TGA curves of CaP068, CaP078 and CaP088.

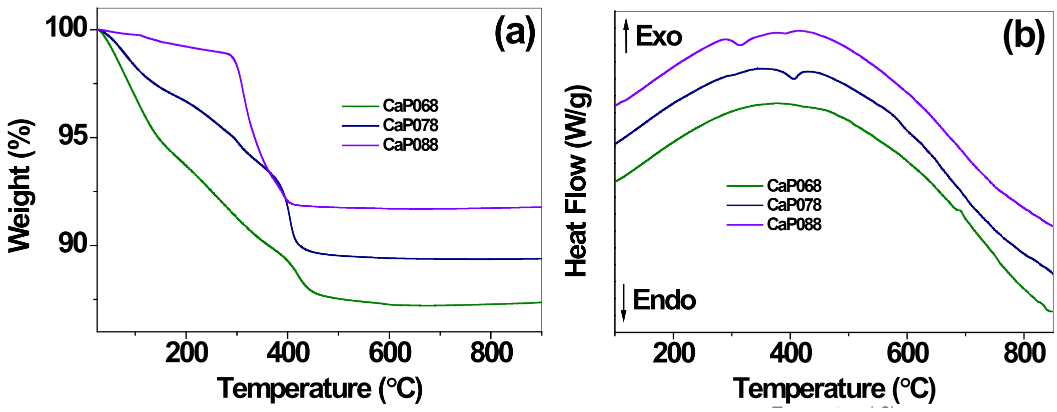

Figure S2. (**a**) Thermogravimetric analysis and (**b**) differential scanning calorimetry heat-flow curves for the calcium phosphate samples CaP068, CaP078 and CaP088.

The carbonate content in ACP032 was calculated as detailed below:

Weight loss up to 300 °C was assumed to be related to the removal of adsorbed or structural water within ACP032. The weight loss between 600 and 800 °C was attributed to the mass of CO2 released from the decomposition of CaCO3 to CaO. This weight loss can be used to back calculate the amount of CaCO3 presence in ACP032. The carbonate content was determined to be ~13.0 wt.% of the starting material (including adsorbed water) and ~15.0 wt.% of the dried ACP032. The calculated carbonate content using this method was close to the value obtained from ICP-OES.

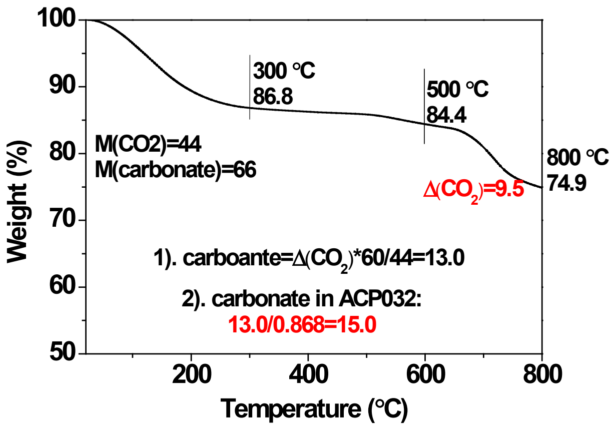

Figure S3. TGA curve of ACP032.

Figure S4 shows the XPS spectra for ACP053 and ACP032. Ca, O, P and C were detected in both samples. The Ca/P atomic ratios in ACP053 and ACP032 were 1.23 and 2.23, respectively, which was in agreement with the ICP-OES results. Three different kinds of carbon were detected in the high-energy resolution XPS C1 s spectrum for ACP032: C-C/C=C at 284.8 eV, adventitious carbon at 287.1 eV and carbon in CO32- at 289.2 eV. However, in ACP032, only C-C at 284.8 eV and carbon in CO32- at 289.9 eV were detected. It should be noted that the relative intensity of carbon in CO32- was much lower in ACP053 than in ACP032 because of the low CO32- content in ACP053. The high-energy resolution XPS O 1s spectrum for ACP053 suggested that O existed in PO43- at 531.2 eV and H2O at 533.5 eV while, in the spectrum for ACP032, O was in PO43- at 531.2 eV, H2O at 533.1 eV and carbonate at 532.1 eV. The Ca 2p spectrum for ACP053 could be divided into Ca 2p3/2 at 347.3 eV and Ca 2p1/2 at 350.9 eV, similar to the Ca 2p spectrum for ACP032 (Ca 2p3/2 at 347.4 eV and Ca 2p1/2 at 351.0 eV). The P 2p spectrum for ACP053 was located at 133.2 eV with P 2p3/2 at 132.9 eV and P 2p1/2 at 133.9 eV while that for ACP032 was at 133.4 eV with P 2p3/2 at 133.0 eV and P 2p1/2 at 133.9 eV.

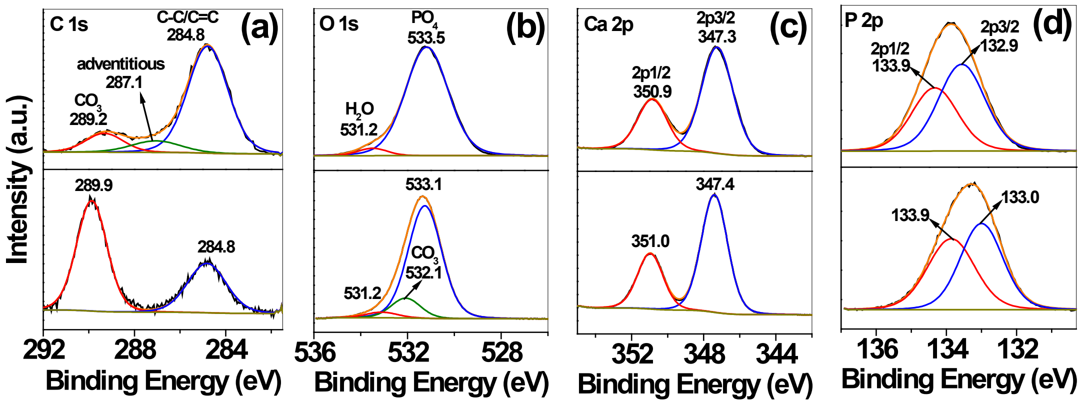

Figure S4. High-resolution X-ray photoelectron spectroscopy C 1s (**a**), O 1s (**b**), Ca 2p (**c**) and P 2p (**d**) spectra for ACP053 (upper panel) and ACP032 (lower panel).

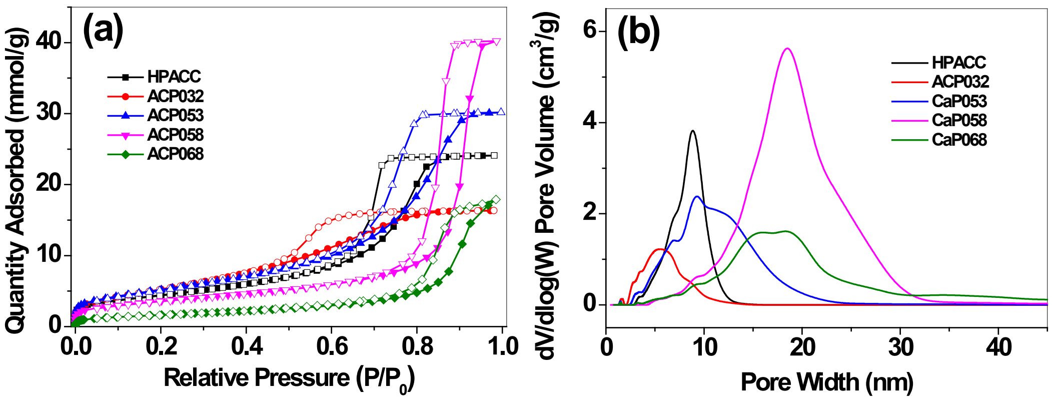

Figure S5. (**a**) N2 adsorption-desorption isotherms and (**b**) density functional theory pore-size distribution curves for the amorphous calcium phosphate (ACP) and calcium phosphate (CaP) samples investigated in this study.

Table S1. Comparison of the synthesis methods, particle sizes, Brunauer–Emmett–Teller (BET) surface areas, pore volumes, pore sizes and applications of amorphous calcium phosphate (ACP) samples synthesized in this work and other ACP and crystalline calcium phosphate (CaP) samples reported in the literature. ACMP = amorphous calcium magnesium phosphate; DCPA = dicalcium phosphate anhydrous; HA = hydroxyapatite; HA-NRMs = HA nanorod-assembled hierarchical microspheres; HA-NRHMs = HA nanorod-assembled hierarchical hollow microspheres.

Samples	Synthesis method	Particle size (nm)	BET (m^2/g)	Pore volume (cm^3/g)	Pore size (nm)	Applications	Ref.
**Amorphous calcium carbonate (ACP)**							
ACP053	Organic solvent based (methanol)	< 10 nm	418	1.05	9.31	Drug delivery	**This work**
Eu doped ACP	microwave-assisted solvothermal	200-300 nm	253-315	1.11-1.39	10.1-15.6	Drug delivery	[8]
ACP microspheres	microwave-assisted hydrothermal	~ hundreds nm	232.9	-	9.9	Drug delivery	[9]
ACP	microwave-assisted solvothermal	~ hundreds nm	209	1.12	17.8	Drug delivery	[8]
ACP nanocomposites	aqueous solution based	~100–200 nm	163	0.37	7–9	Enzyme loading	[10]
ACP spherules	aqueous solution based	10.8 ± 1.4 nm	150-170	-	-	-	[11]
ACP nanoparticles	aqueous solution based	14-16 nm	133-154	-	-	-	[12]
ACP nanowires	microwave-assisted hydrothermal	-	115	-	24	Metal ion adsorption	[13]
ACP ($CaHPO_4$)	organic solvent based (anhydrous ethanol)	60-80nm	123	-	-	-	[14]
Sr-ACP porous microspheres	microwave-hydrothermal	~hundreds nm	110.1	0.69	17.2	Drug delivery; Bone regeneration	[15]

La-ACP nanospheres	Aqueous solution based	10-30 nm	108.3	1.3	-	Drug delivery	[16]
ACP porous microspheres	microwave-hydrothermal	~hundreds nm	106.7	1.02	25.7	Drug delivery; Bone regeneration	[15]
ACP vesicle-like nanospheres	sonochemical synthesis (water and EG)	150–240 nm	93.1	0.96	30.2	Drug delivery	[17]
ACP nanospheres	mixing solution	10-30 nm	77.0	1.0	-	Protein delivery	[16]
ACP mesoporous microspheres	microwave-assisted hydrothermal	681 nm	67.1	0.23	7.6	Drug delivery	[18]
ACP	aqueous solution based	-	50-60	-	-	-	[19]
ACP-phosphorylated microspheres	hydrothermal (water-ethanol)	~750 nm	55.2	-	5.2	Drug delivery	[20]
ACP microspheres	microwave-assisted hydrothermal	330 ±135 nm	23.8	-	16.9 nm	Drug delivery	[21]
ACMP	microwave hydrothermal	~hundreds nm	5.90	0.03	< 9 nm	Drug delivery	[22]
**Crystalline calcium phosphate (CaP)**							
Hydroxyapatite nanoparticles	aqueous solution based- mixed surfactant system	4-20 nm	364	-	-	-	[23]
Nanocrystalline Apatite	aqueous solution based	Wide: 1.5-4 nm Long: 70-90 nm	356	-	-	-	[11]
carbonated HA	aqueous solution based	length: 15-20 nm; width: 3-5 nm	300 ±10	0.46	0.48; 4.2	-	[24]
CaP particles -F127	aqueous solution based	30-80 nm	263 ± 36	0.96 ± 0.14	13.54 ± 3.52	Protein delivery	[25]
Sm^{3+}-Fe^{3+} co-doped HA	aqueous solution based	length: 200~350 nm diameter: 50~100 nm	153.52	0.3286	3.64 and 9.01 nm	Drug delivery	[26]
HA with fish sperm DNA	microwave-hydrothermal	~hundreds nm	102.0	1.02	30.65	Protein delivery	[27]
HA nanorods	microwave-hydrothermal	~hundred nm	85.8	0.98	32.3	Drug delivery; Bone regeneration	[15]
DCPA particles	microwave solvent thermal (ethanol)	50–110 nm × 40–90 nm	77.8	-	4-12	Protein delivery	[28]
HA (pH10)	microwave-hydrothermal	~hundreds nm	71.0	0.83	26.7	Protein release	[27]
HA nanorods (HA-NRHMs)	microwave-assisted heating	<1 μm	51.6	-	17.2	Drug delivery	[29]

beta-tricalcium phosphate	microwave solvent thermal (methanol)	20–80 nm × 10–20 nm	34.4	-	10-40	Protein delivery	[28]
CaP microflowers	Aqueous solution based	2.70 ± 0.22 µm	21.55	-	15.9	Bone regeneration	[30]
HA nanorods (HA-NRMs)	microwave-assisted hydrothermal	14 to 20 µm	16.3	-	14.4	Drug Delivery	[29]
HA particles	hydrothermal	1–5 µm × 0.4–1.2 µm	16.1	0	-	Protein Delivery	[28]

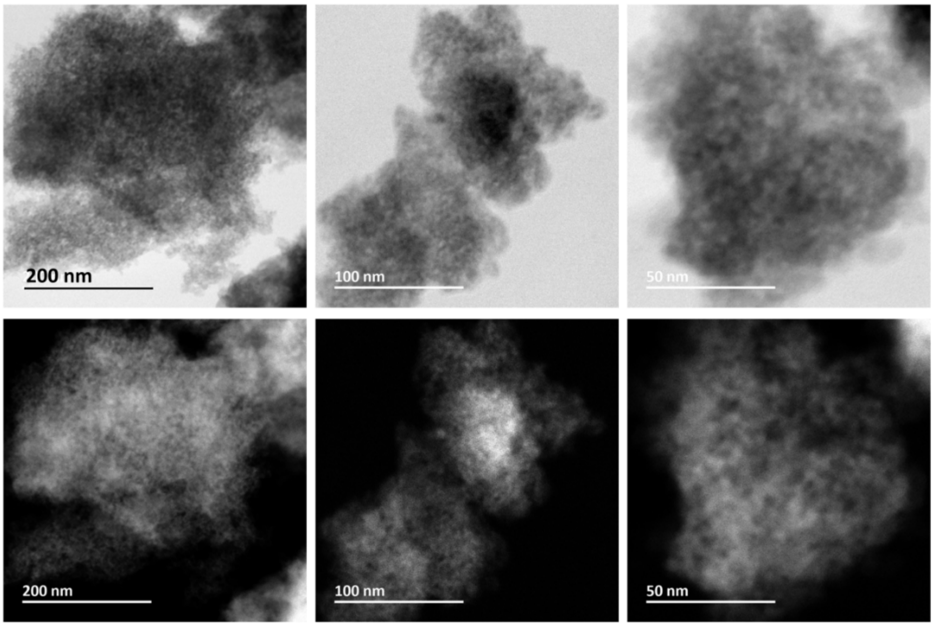

Figure S6. Bright-field (upper panel) and high-angle annular dark-field (lower panel) scanning transmission electron microscopy images of the amorphous calcium phosphate sample ACP053.

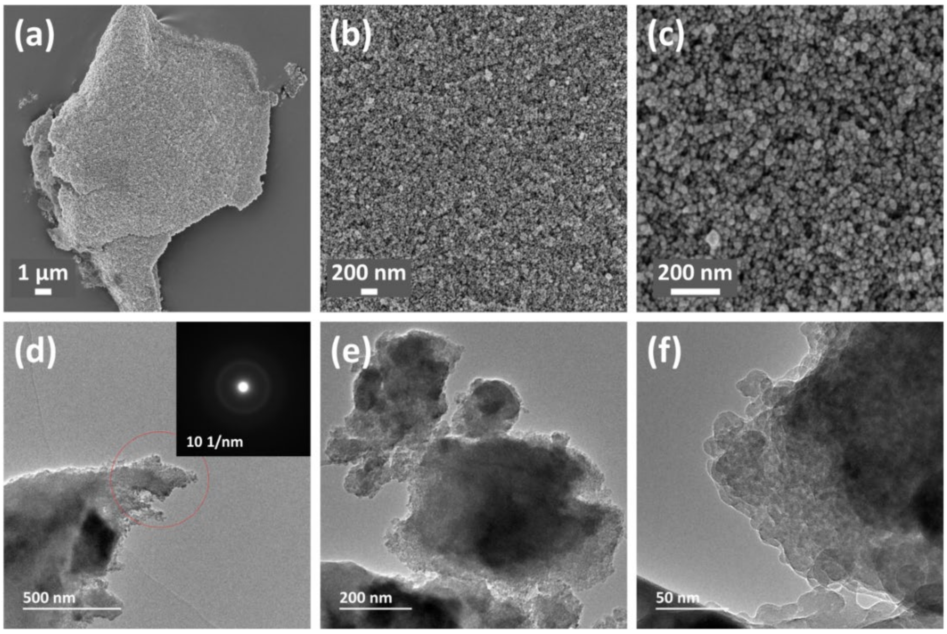

Figure S7. Scanning electron microscopy (**a**-**c**) and transmission electron microscopy (**d**-**f**) images of the amorphous calcium phosphate sample ACP032 (the insert in Figure S7d is the corresponding selected-area electron diffraction pattern).

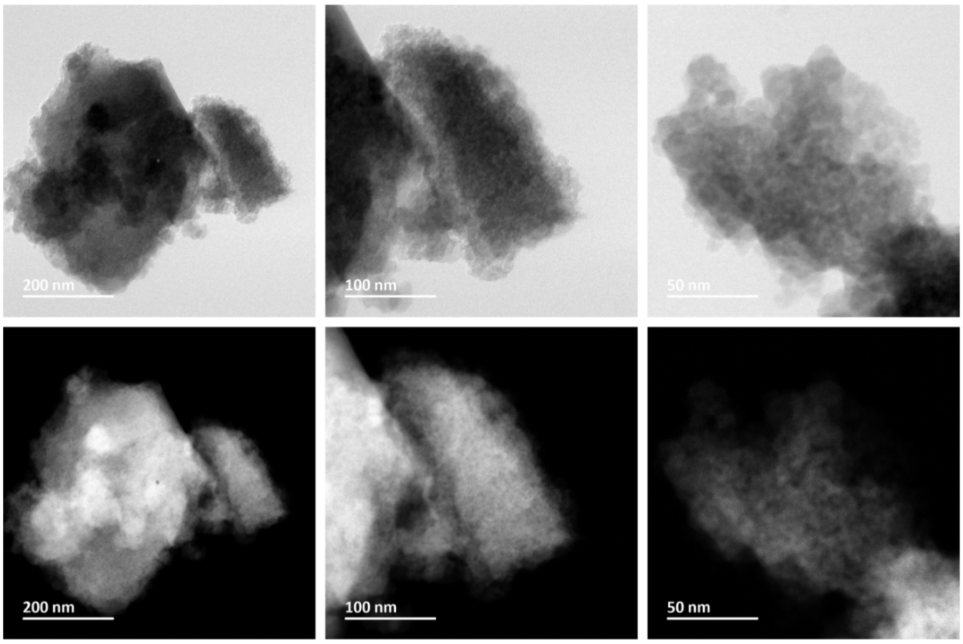

Figure S8. Bright-field (upper panel) and high-angle annular dark-field (lower panel) scanning transmission electron microscopy images of the amorphous calcium phosphate sample ACP032.

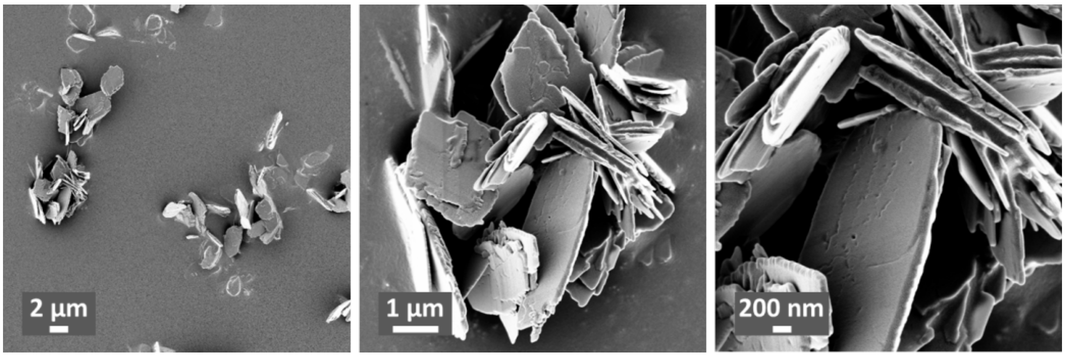

Figure S9. Scanning electron microscopy images of the calcium phosphate sample CaP088.

S3. Stability of the ACPs

S3.1. Stability in air

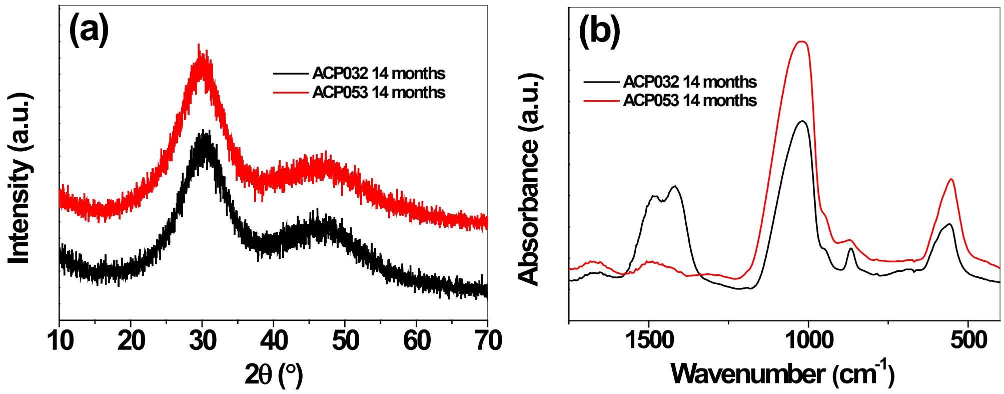

Figure S10. (**a**) Powder X-ray diffraction patterns and (**b**) infrared spectra for amorphous calcium carbonate samples ACP053 and ACP032 after storage under ambient conditions for 14 months.

Table S2. Comparison of the stability of amorphous calcium carbonate (ACP) samples synthesized in this work or reported in the literature. Italics denote that the ACPs were synthesized with a stabilizer. ACMP = amorphous calcium magnesium phosphate.

Sample	Stability in air	Stability in solution (dried ACP soaked in solution)			Ref.
		water	phosphate buffered saline	other media	
ACP053	> 14 months	~180 min	-	cell culture medium pH=7.4: ~24 hours	this work
ACP032	> 14 months	~1800 min	-	cell culture medium pH=7.4: >48 hours	this work
ACP synthesized at pH=9	<= 5months	-	-	-	[12]
ACP synthesized at pH=10/11	>7 months	-	-	-	[12]
ACP	wet atmosphere saturated in water vapor: 40 °C, 6 hours 60 °C , 1 hour 80 °C, 30 min	-	-	-	[31]
ACP		-	-	buffer solution: pH=7, 6 min pH=8, 14 min pH=9, 35 min pH=10, 45 min	[32]
ACP-poly(d,l-lactic acid) composite	-	-	-	simulated body fluid: pH=7.4, < 1 day	[33]
citrate-stabilized ACP		*830 min*	*pH=7.3±0.01, 10 min*	-	*[34]*
vesicle-like ACP	-	-	*pH=4.5, >300 hours pH=7.4, >300 hours*	-	*[17]*
ACP nanospheres			*pH=4.5, >150 hours pH=7.4, >150 hours*	-	*[35]*
ACMP	-	-	*pH=7.4, >190 hours*	*physiological saline: >190 hours*	*[22]*

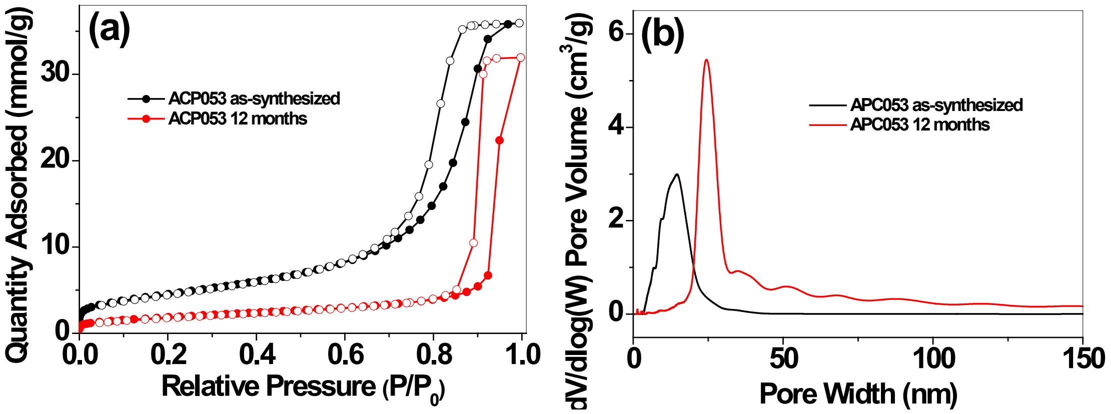

Figure S11. (**a**) N2 adsorption/desorption isotherms and (**b**) density functional theory pore-size distribution curves for the amorphous calcium carbonate sample ACP053 as synthesized and after storage under ambient conditions for 12 months.

S3.2. Stability in de-ionized water

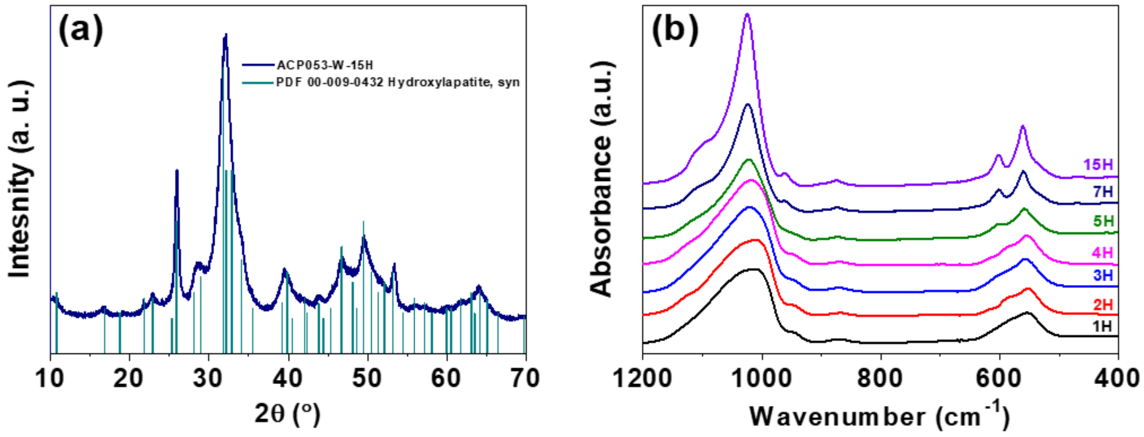

Figure S12. (**a**) Powder X-ray diffraction pattern for the amorphous calcium carbonate sample ACP053 after exposure to de-ionized water for 15 hours (donated as ACP053-W-15H), also showing the diffraction peaks for hydroxyapatite (PDF 00-009-0432) and (**b**) infrared spectra for ACP053 after immersion in de-ionized water for 1-15 hours.

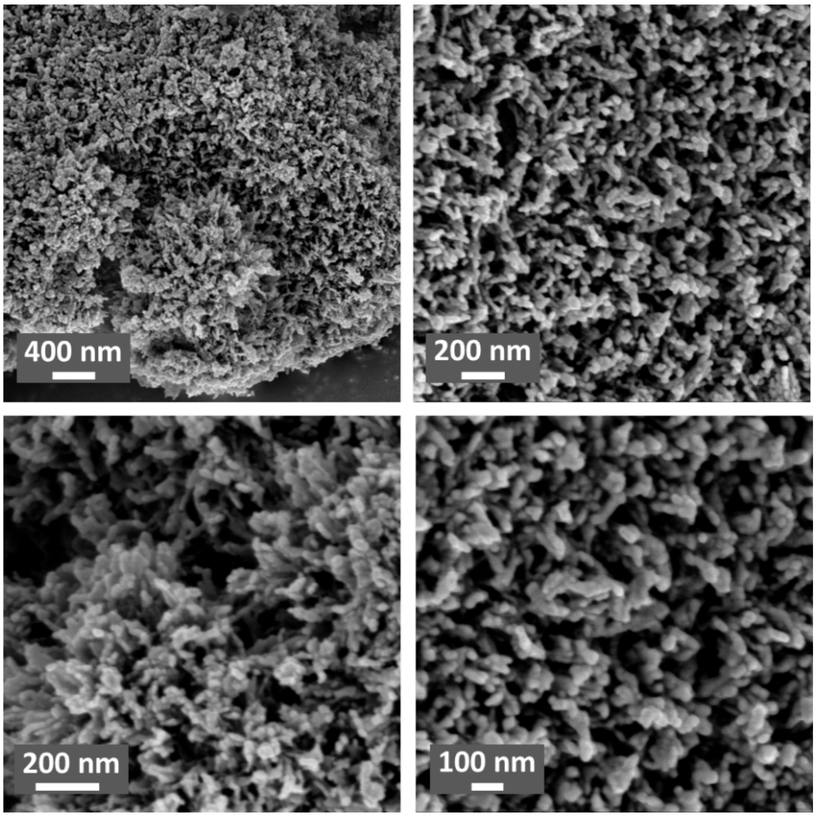

Figure S13. Scanning electron microscopy images of the hydroxyapatite formed from amorphous calcium phosphate sample ACP053 after exposure to de-ionized water for 15 hours.

The stability of ACP032 after exposure to de-ionized water was explored using the same methods as for ACP053. Infrared (IR) spectra for ACP032 after exposure to de-ionized water for 1 hour to 50 days are shown in Figure 14a. The spectrum for ACP032 after exposure to water for 20 hours had the ν2 band for CO32- at around 866.6 cm-1 and the ν4 band for PO43- at 582.5 cm-1 and 560.6 cm-1, similar to those for as-synthesized ACP032, indicating that it had remained amorphous for over 20 hours. After exposure to de-ionized water for 30 hours, the ν2 band for CO32- became sharp and shifted to ~870 cm-1, which suggested that ACP032 had started to crystallize. The PXRD pattern confirmed that calcite had formed at this stage (Figure S15). The ν4 band for PO43- began to split into individual bands after ACP032 had been immersed in de-ionized water for 15 days. The bands became well-defined after 30 days. These bands were attributed to HA, which suggested that ACP032 had crystallized to HA after exposure to de-ionized water for 15 days and that this crystallization process proceeded over 30 days. The IR spectra for ACP032 after exposure to de-ionized water for 1 hour and 30 days, and for calcite and HA are shown in Figure S14b. The ν4 and ν2 bands for CO32- and the ν4 band for PO43- were used to follow the crystallization process of ACP032. The PXRD pattern for ACP032 after exposure to de-ionized water for 30 days demonstrated that the formed crystalline phases were calcite (PDF 00-002-0623) and HA (PDF 00-001-1008 and PDF 00-066-0147), as shown in Figure S15b.

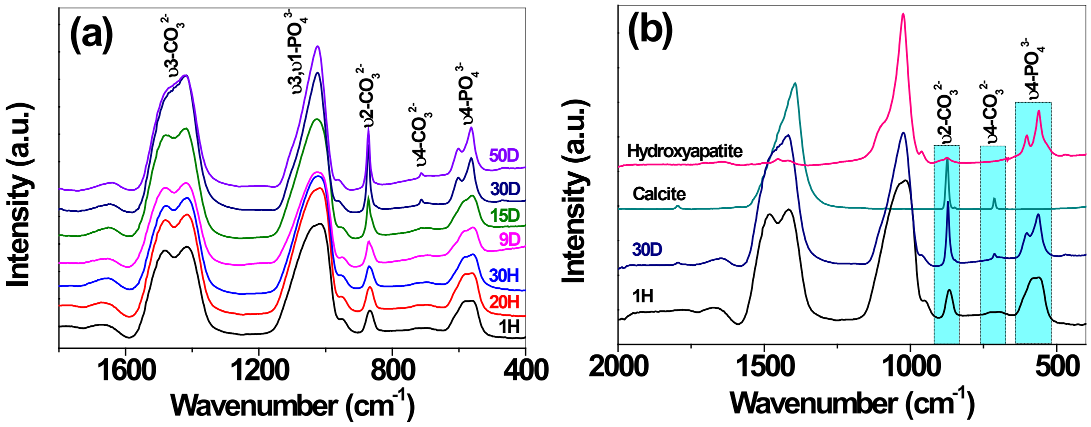

Figure S14. (**a**) Infrared (IR) spectra for the amorphous calcium phosphate sample ACP032 after exposure to de-ionized water for 1 hour to 50 days and (**b**) IR spectra for ACP032 after exposure to de-ionized water for 1 hour and 30 days, in comparison to those for hydroxyapatite and calcite.

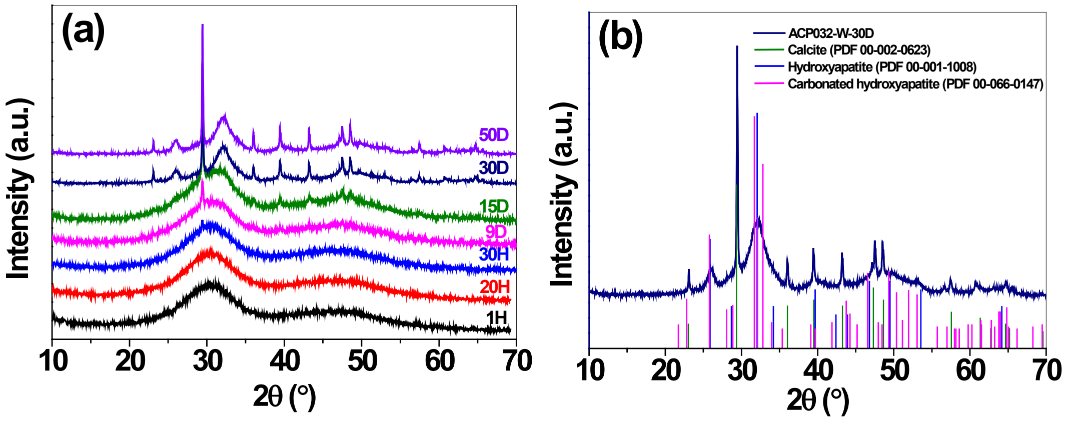

Figure S15. Powder X-ray diffraction patterns for the amorphous calcium phosphate sample ACP032 after exposure to de-ionized water for (**a**) 1 hour to 50 days and (**b**) 30 days (ACP032-W-30D), showing the diffraction peaks for calcite (PDF 00-002-0623), hydroxyapatite (PDF 00-001-1008) and carbonated hydroxyapatite (PDF 00-066-0147).

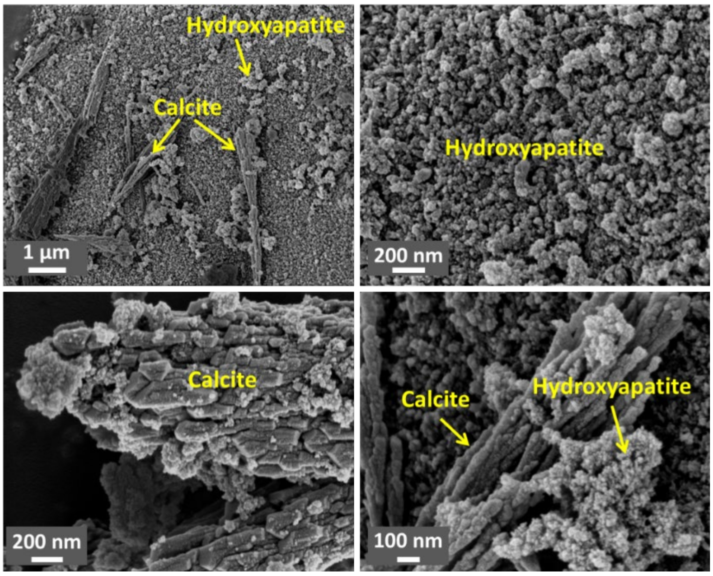

Figure S16. Scanning electron microscopy images of the amorphous calcium phosphate sample ACP032 after exposure to de-ionized water for 50 days.

S3.3. Stability in cell culture medium

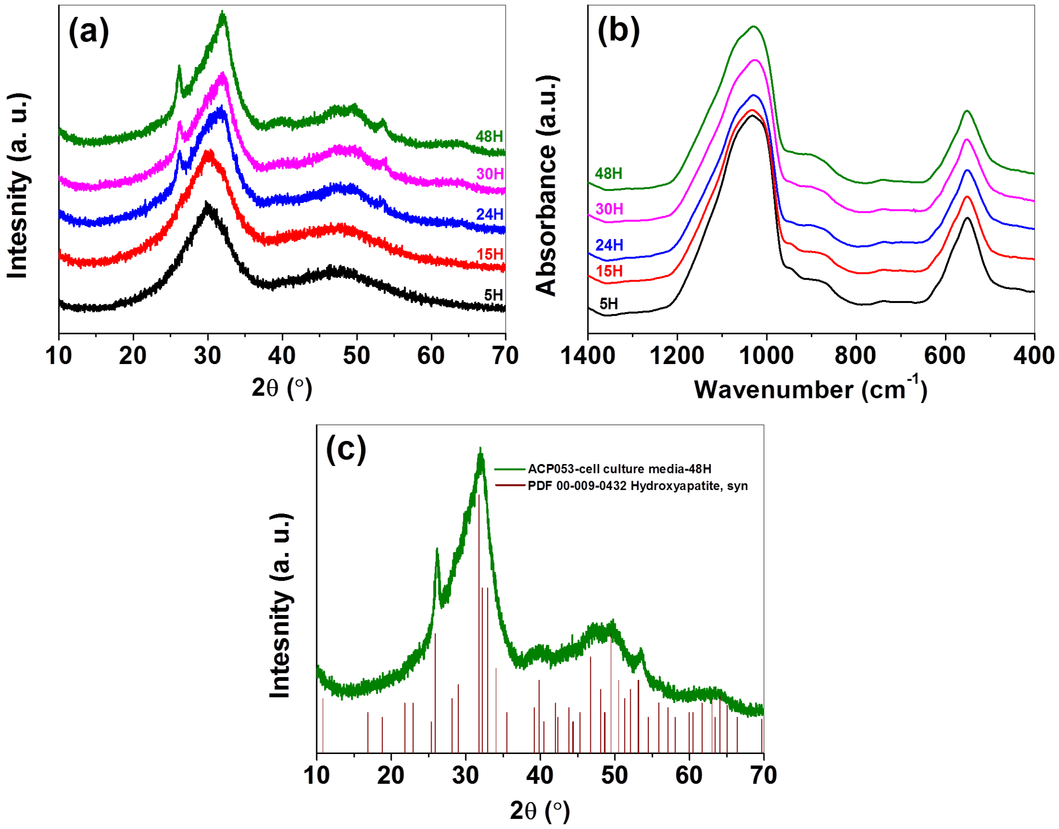

Figure S17. (**a**) Powder X-ray diffraction (PXRD) patterns and (**b**) infrared spectra for the amorphous calcium phosphate sample ACP053 after exposure to cell culture medium for 5-48 hours. (**c**) PXRD pattern for ACP053 after exposure to cell culture medium for 48 hours, showing the diffraction peaks for hydroxyapatite (PDF 00-009-0432).

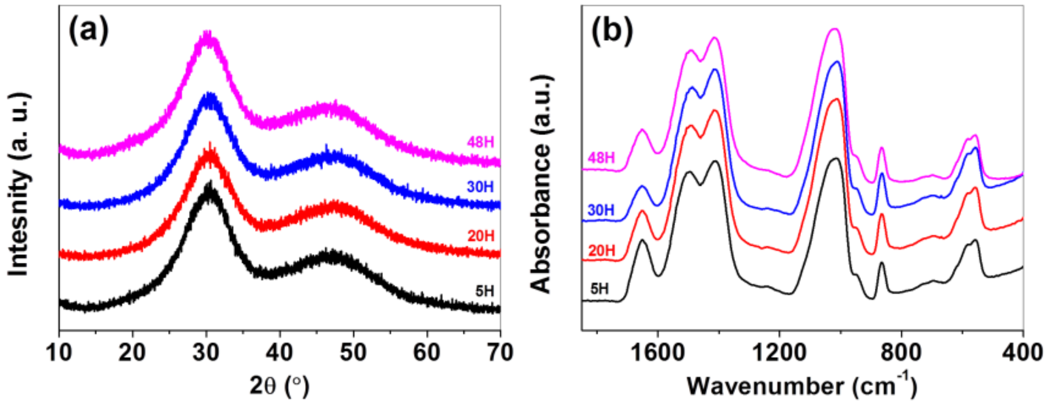

Figure S18. (**a**) Powder X-ray diffraction patterns and (**b**) infrared spectra for the amorphous calcium phosphate sample ACP032 after exposure to cell culture medium for 5-48 hours.

S4. acp053 used as a drug carrier

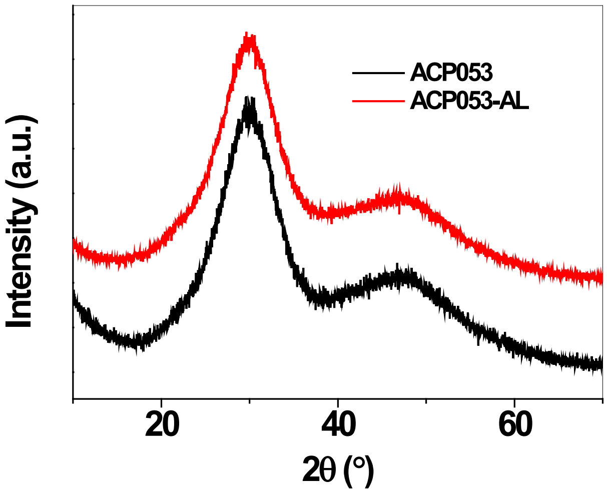

Figure S19. Powder X-ray diffraction patterns for the amorphous calcium phosphate sample ACP053 before and after loading with alendronate (AL).

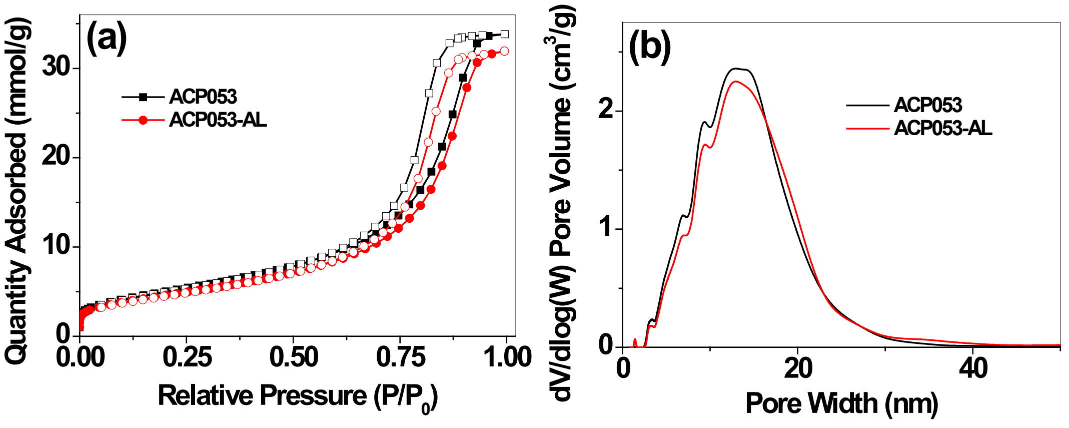

Figure S20. (**a**) N2 sorption isotherms for the amorphous calcium phosphate sample ACP053 before and after loading with alendronate (AL) and (**b**) the corresponding density functional theory pore-size distribution graphs.

Table S3. Porous properties of ACP053 and AL loaded ACP053-AL

	Porous ACP	ACP-AL
ACP content (g)	1.0	0.944
Drug content (g)	0	0.056
Avalable overall pore volume (cm^3/g)	1.17	1.10
Specific density of the loaded drug (g/cm^3)	-	1.7180
Volume of the loaded drug (cm^3)	-	0.03
Expected pore volume (cm^3/g)	-	1.07
Pore volume from N_2 adsorption (cm^3/g)	-	1.11

Reference in Supporting Information

1. Sun, R., Zhang, P., Bajnóczi, É. G., Neagu, A., Tai, C.-W., Persson, I., Strømme, M. and Cheung, O. Amorphous Calcium Carbonate Constructed from Nanoparticle Aggregates with Unprecedented Surface Area and Mesoporosity. *ACS Appl. Mater. Interfaces* **2018,** *10*, 21556-21564.
2. Taha, E. A. and Youssef, N. F. Spectrophotometric determination of some drugs for osteoporosis. *Chem. Pharm. Bull.* **2003,** *51*, 1444-1447.
3. Bushra, M. U., Akter, N., Hassan, M. R., Islam, A. and Hossain, M. R. Development and validation of a simple UV spectrophotometric method for the determination of cefotaxime sodium in bulk and pharmaceutical formulation. *IOSR J. Pharm* **2014,** *4*, 74-77.
4. Nafea, E. H., El-Massik, M. A., El-Khordagui, L. K., Marei, M. K. and Khalafallah, N. M. Alendronate PLGA microspheres with high loading efficiency for dental applications. *J. Microencapsulation* **2007,** *24*, 525-538.
5. Long, K. A., Jackson, J. K., Yang, C., Chehroudi, B., Brunette, D. M. and Burt, H. M. Controlled release of alendronate from polymeric films. *Journal of Biomaterials Science, Polymer Edition* **2009,** *20*, 653-672.
6. Palazzo, B., Iafisco, M., Laforgia, M., Margiotta, N., Natile, G., Bianchi, C. L., Walsh, D., Mann, S. and Roveri, N. Biomimetic hydroxyapatite–drug nanocrystals as potential bone substitutes with antitumor drug delivery properties. *Adv. Funct. Mater.* **2007,** *17*, 2180-2188.
7. Zyman, Z., Epple, M., Goncharenko, A., Rokhmistrov, D., Prymak, O. and Loza, K. Thermally induced crystallization and phase evolution in powders derived from amorphous calcium phosphate precipitates with a Ca/P ratio of 1: 1. *J. Cryst. Growth* **2016,** *450*, 190-196.
8. Chen, F., Huang, P., Qi, C., Lu, B.-Q., Zhao, X.-Y., Li, C., Wu, J., Cui, D.-X. and Zhu, Y.-J. Multifunctional biodegradable mesoporous microspheres of Eu 3+-doped amorphous calcium phosphate: microwave-assisted preparation, pH-sensitive drug release, and bioimaging application. *J. Mater. Chem. B* **2014,** *2*, 7132-7140.
9. Ding, G.-J., Zhu, Y.-J., Qi, C., Lu, B.-Q., Wu, J. and Chen, F. Porous microspheres of amorphous calcium phosphate: block copolymer templated microwave-assisted hydrothermal synthesis and application in drug delivery. *J. Colloid Interface Sci.* **2015,** *443*, 72-79.
10. Hwang, E. T., Tatavarty, R., Chung, J. and Gu, M. B. New functional amorphous calcium phosphate nanocomposites by enzyme-assisted biomineralization. *ACS Appl. Mater. Interfaces* **2013,** *5*, 532-537.
11. He, W., Kjellin, P., Currie, F., Handa, P., Knee, C. S., Bielecki, J., Wallenberg, L. R. and Andersson, M. Formation of bone-like nanocrystalline apatite using self-assembled liquid crystals. *Chem. Mater.* **2011,** *24*, 892-902.
12. Vecstaudza, J. and Locs, J. Novel preparation route of stable amorphous calcium phosphate nanoparticles with high specific surface area. *J. Alloys Compd.* **2017,** *700*, 215-222.
13. Ding, G.-J., Zhu, Y.-J., Qi, C., Sun, T.-W., Wu, J. and Chen, F. Amorphous calcium phosphate nanowires prepared using beta-glycerophosphate disodium salt as an organic phosphate source by a microwave-assisted hydrothermal method and adsorption of heavy metals in water treatment. *RSC Adv.* **2015,** *5*, 40154-40162.
14. Layrolle, P. and Lebugle, A. Characterization and reactivity of nanosized calcium phosphates prepared in anhydrous ethanol. *Chem. Mater.* **1994,** *6*, 1996-2004.
15. Yu, W., Sun, T.-W., Qi, C., Ding, Z., Zhao, H., Chen, F., Chen, D., Zhu, Y.-J., Shi, Z. and He, Y. Strontium-doped amorphous calcium phosphate porous microspheres synthesized through a microwave-hydrothermal method using fructose 1, 6-bisphosphate as an organic phosphorus source: application in drug delivery and enhanced bone regeneration. *ACS Appl. Mater. Interfaces* **2017,** *9*, 3306-3317.
16. Chen, F., Yang, B., Qi, C., Sun, T.-W., Jiang, Y.-Y., Wu, J., Chen, X. and Zhu, Y.-J. An amorphous calcium phosphate nanocomposite for storing and sustained release of IgY protein with antibacterial activity. *RSC Adv.* **2015,** *5*, 100682-100688.
17. Qi, C., Zhu, Y.-J., Zhang, Y.-G., Jiang, Y.-Y., Wu, J. and Chen, F. Vesicle-like nanospheres of amorphous calcium phosphate: sonochemical synthesis using the adenosine 5′-triphosphate disodium salt and their application in pH-responsive drug delivery. *J. Mater. Chem. B* **2015,** *3*, 7347-7354.
18. Qi, C., Zhu, Y. J., Sun, T. W., Wu, J. and Chen, F. Microwave-Assisted Hydrothermal Rapid Synthesis of Amorphous Calcium Phosphate Mesoporous Microspheres Using Adenosine 5′-Diphosphate and Application in pH-Responsive Drug Delivery. *Chem. Asian J.* **2015,** *10*, 2503-2511.

19. Holmes, J. M. and Beebe, R. A. Surface areas by gas adsorption on amorphous calcium phosphate and crystalline hydroxyapatite. *Calcif. Tissue Int.* **1971,** *7*, 163-174.
20. Zhou, Z.-F., Sun, T.-W., Chen, F., Zuo, D.-Q., Wang, H.-S., Hua, Y.-Q., Cai, Z.-D. and Tan, J. Calcium phosphate-phosphorylated adenosine hybrid microspheres for anti-osteosarcoma drug delivery and osteogenic differentiation. *Biomaterials* **2017,** *121*, 1-14.
21. Ding, G.-J., Zhu, Y.-J., Qi, C., Lu, B.-Q., Chen, F. and Wu, J. Porous hollow microspheres of amorphous calcium phosphate: soybean lecithin templated microwave-assisted hydrothermal synthesis and application in drug delivery. *J. Mater. Chem. B* **2015,** *3*, 1823-1830.
22. Qi, C., Zhu, Y.-J., Chen, F. and Wu, J. Porous microspheres of magnesium whitlockite and amorphous calcium magnesium phosphate: microwave-assisted rapid synthesis using creatine phosphate, and application in drug delivery. *J. Mater. Chem. B* **2015,** *3*, 7775-7786.
23. Uota, M., Arakawa, H., Kitamura, N., Yoshimura, T., Tanaka, J. and Kijima, T. Synthesis of high surface area hydroxyapatite nanoparticles by mixed surfactant-mediated approach. *Langmuir* **2005,** *21*, 4724-4728.
24. Padilla, S., Izquierdo-Barba, I. and Vallet-Regí, M. High specific surface area in nanometric carbonated hydroxyapatite. *Chem. Mater.* **2008,** *20*, 5942-5944.
25. Ng, S., Guo, J., Ma, J. and Loo, S. C. J. Synthesis of high surface area mesostructured calcium phosphate particles. *Acta Biomater.* **2010,** *6*, 3772-3781.
26. Haifeng, G., Zhiqiang, Z., Feng, Y., Guoping, L. and Zhiheng, Z. Preparation of magnetic, luminescent and mesoporous hydroxyapatite nanospindles with high specific surface area. *Rare Metal Mater. Eng.* **2014,** *43*, 2647-2651.
27. Chen, X., Yang, B., Qi, C., Sun, T.-W., Chen, F., Wu, J., Feng, X.-P. and Zhu, Y.-J. DNA-templated microwave-hydrothermal synthesis of nanostructured hydroxyapatite for storing and sustained release of an antibacterial protein. *Dalton Trans.* **2016,** *45*, 1648-1656.
28. Ching Lau, C., Reardon, P. J. T., Campbell Knowles, J. and Tang, J. Phase-tunable calcium phosphate biomaterials synthesis and application in protein delivery. *ACS Biomaterials Sci. Eng.* **2015,** *1*, 947-954.
29. Zhao, X. Y., Zhu, Y. J., Qi, C., Chen, F., Lu, B. Q., Zhao, J. and Wu, J. Hierarchical Hollow Hydroxyapatite Microspheres: Microwave-Assisted Rapid Synthesis by Using Pyridoxal-5′-Phosphate as a Phosphorus Source and Application in Drug Delivery. *Chem. Asian J.* **2013,** *8*, 1313-1320.
30. Tian, T., Liao, J., Zhou, T., Lin, S., Zhang, T., Shi, S.-R., Cai, X. and Lin, Y. Fabrication of calcium phosphate microflowers and their extended application in bone regeneration. *ACS Appl. Mater. Interfaces* **2017,** *9*, 30437-30447.
31. Rodrigues, A. and Lebugle, A. Behavior in wet atmosphere of an amorphous calcium phosphate with an atomic Ca/P ratio of 1.33. *J. Solid State Chem.* **1999,** *148*, 308-315.
32. Boskey, A. L. and Posner, A. S. Conversion of amorphous calcium phosphate to microcrystalline hydroxyapatite. A pH-dependent, solution-mediated, solid-solid conversion. *J. Phys. Chem.* **1973,** *77*, 2313-2317.
33. Zhang, H., Fu, Q.-W., Sun, T.-W., Chen, F., Qi, C., Wu, J., Cai, Z.-Y., Qian, Q.-R. and Zhu, Y.-J. Amorphous calcium phosphate, hydroxyapatite and poly (D, L-lactic acid) composite nanofibers: electrospinning preparation, mineralization and in vivo bone defect repair. *Colloids Surf. B: Biointerfaces* **2015,** *136*, 27-36.
34. Chatzipanagis, K., Iafisco, M., Roncal-Herrero, T., Bilton, M., Tampieri, A., Kröger, R. and Delgado-López, J. M. Crystallization of citrate-stabilized amorphous calcium phosphate to nanocrystalline apatite: a surface-mediated transformation. *CrystEngComm* **2016,** *18*, 3170-3173.
35. Qi, C., Zhu, Y. J., Zhao, X. Y., Lu, B. Q., Tang, Q. L., Zhao, J. and Chen, F. Highly stable amorphous calcium phosphate porous nanospheres: microwave-assisted rapid synthesis using ATP as phosphorus source and stabilizer, and their application in anticancer drug delivery. *Chem. Eur. J.* **2013,** *19*, 981-987.
